# Sexually dimorphic extracellular vesicle responses after chronic spinal cord injury are associated with neuroinflammation and neurodegeneration in the aged brain

**DOI:** 10.1186/s12974-023-02881-z

**Published:** 2023-08-31

**Authors:** Yun Li, Niaz Khan, Rodney M. Ritzel, Zhuofan Lei, Samantha Allen, Alan I. Faden, Junfang Wu

**Affiliations:** Department of Anesthesiology and Center for Shock, Trauma and Anesthesiology Research (STAR), University of Maryland School of Medicine, 685 W. Baltimore Street, MSTF, Room 6-034D, Baltimore, MD 21201 USA

**Keywords:** Chronic spinal cord injury, Extracellular vesicles, Biological sex, Brain transcriptional changes, microRNAs, EVs content, Neuroinflammation

## Abstract

**Background:**

Medical advances have made it increasingly possible for spinal cord injury (SCI) survivors to survive decades after the insult. But how SCI affects aging changes and aging impacts the injury process have received limited attention. Extracellular vesicles (EVs) are recognized as critical mediators of neuroinflammation after CNS injury, including at a distance from the lesion site. We have previously shown that SCI in young male mice leads to robust changes in plasma EV count and microRNA (miR) content. Here, our goal was to investigate the impact of biological sex and aging on EVs and brain after SCI.

**Methods:**

Young adult age-matched male and female C57BL/6 mice were subjected to SCI. At 19 months post-injury, total plasma EVs were isolated by ultracentrifugation and characterized by nanoparticle tracking analysis (NTA). EVs miR cargo was examined using the Fireplex® assay. The transcriptional changes in the brain were assessed by a NanoString nCounter Neuropathology panel and validated by Western blot (WB) and flow cytometry (FC). A battery of behavioral tests was performed for assessment of neurological function.

**Results:**

Transcriptomic changes showed a high number of changes between sham and those with SCI. Sex-specific changes were found in transcription networks related to disease association, activated microglia, and vesicle trafficking. FC showed higher microglia and myeloid counts in the injured tissue of SCI/Female compared to their male counterparts, along with higher microglial production of ROS in both injured site and the brain. In the latter, increased levels of TNF and mitochondrial membrane potential were seen in microglia from SCI/Female. WB and NTA revealed that EV markers are elevated in the plasma of SCI/Male. Particle concentration in the cortex increased after injury, with SCI/Female showing higher counts than SCI/Male. EVs cargo analysis revealed changes in miR content related to injury and sex. Behavioral testing confirmed impairment of cognition and depression at chronic time points after SCI in both sexes, without significant differences between males and females.

**Conclusions:**

Our study is the first to show sexually dimorphic changes in brain after very long-term SCI and supports a potential sex-dependent EV-mediated mechanism that contributes to SCI-induced brain changes.

**Supplementary Information:**

The online version contains supplementary material available at 10.1186/s12974-023-02881-z.

## Introduction

The long-term survival rate following traumatic spinal cord injury (SCI) is significantly prolonged with assistive technology, rehabilitative interventions, and the ability to identify and intervene in secondary conditions [1–3], some even into their seventh or eighth decade [4–6]. These survival changes have led neurotrauma researchers to examine how SCI affects the aging process and how aging alters the trajectory of SCI outcome. Patients with long-term SCI can have lower life expectancy and quality-of-life, along with higher risk of comorbidities and complications [6, 7]. One of the long-term consequences is neuropsychological impairment, which includes deficits in memory, executive functions, attention span, processing speed and learning abilities [8–10]. Clinical data indicate that the risk of chronic SCI patients developing cognitive impairment and dementia is 13 times higher than similar aged individuals without injury [9, 11, 12]. We and others have shown that neuropsychological impairment is caused, in part, by neuroinflammation in the brain, and that SCI-induced depression and cognitive dysfunction reflect discrete neuropathological changes [13–21]. But how proinflammatory factors and signaling from the injury site in the spinal cord reach to distal brain regions have not been well elucidated.

Among potential mechanisms leading to pathological changes in distal brain regions after SCI is circulating extracellular vesicles (EVs) [22]. EVs are membranous vesicles enclosed by a lipid bilayer that can be secreted by most eukaryotic cells and contain biologically active cargo from their cells of origin. EVs are known to play critical roles in intercellular communication by transferring bioactive components, such as messenger RNAs, microRNAs (miRs), genomic DNA and peptides or proteins to recipient cells in the body [23]. Several recent articles have found that EVs can be used as informative indicators of the endosomal–lysosomal pathway, with changes occurring in multiple neurodegenerative diseases, such as Alzheimer’s disease (AD) and Parkinson disease (PD) [24, 25]. Our previous study characterized EVs dynamics at acute and subacute timepoints after injury up to 14d after SCI [26]; however, changes to plasma- and tissue-derived EVs in the chronic post-traumatic period are not well understood.

It had been previously reported by our lab and others that biological sex plays a role in the acute stages and functional recovery process after traumatic SCI [16, 27, 28], with significant sex differences relating to injury-induced neuroinflammation and pain processing [29, 30]. We previously reported significantly less cognitive dysfunction in female mice at 13 weeks post-injury as compared to male mice [16]. How the factors of aging, EVs, and biological sex converge during the long-term recovery process following SCI becomes an intriguing topic to pursue.

In the present study, we subjected age-matched young adult C57BL/6 mice of both sexes to a moderate SCI and explored how biological sex plays a factor in aging with SCI. Using a combination of molecular, cellular, and behavioral techniques, we assessed the functional and pathological outcomes of each sex at 19 months post-injury. Furthermore, we assessed EV count and content using nanoparticle tracking analysis and microRNA multiplex assay at the final endpoint.

## Materials and methods

### Animals and mouse spinal cord contusion model

Young adult age-matched (10–12 weeks) male (M) and female (F) C57BL/6 mice were obtained from Jackson Laboratories. Mice were housed in a controlled environment with 12-h light:dark cycle, with ad libitum food and water provided. All study procedures were performed according to experimental protocols approved by the University of Maryland School of Medicine Institutional Animal Care and Use Committee (IACUC). Spinal cord contusion injury was performed using the Infinite Horizon spinal cord impactor (Precision Systems and Instrumentation) as previously detailed [31, 32]. In brief, induction of anesthesia began at 3% isoflurane and maintained at 1.5% with a nose cone throughout the procedure. After laminectomy, the spinal column was stabilized with bilateral steel clamps and a midline spinal contusion injury was made to the exposed T10 level with a force of 65 kilodynes. Mice in sham groups went through isoflurane anesthesia for the same duration, but did not receive a laminectomy or spinal cord impact. The bladders of injured mice were manually voided by experimenters at least two times per day post-injury until reflex bladder emptying has fully recovered. Experimenters who performed functional assessment and final data analysis were blinded to group designations throughout the process. Because we did not assess the estrous cycle in females, all mice of the female sex were randomly assigned to surgical condition of either sham or SCI with respect to hormonal status. The number of mice used for each experiment is indicated in the figure legends.

### RNA extraction and NanoString neuropathology panel analysis

RNA samples were obtained after tissue processing with the Qiagen RNA extraction kit from the somatosensory cortex (tissue puncta) and the whole hippocampus at 19 months post-injury. Total RNA (20 ng/µl) was run on a NanoString nCounter® system for Mouse Neuropathology v1.0 panel (NanoString Technologies, Seattle, WA) to profile RNA transcript counts for 770 genes and 13 housekeeping genes. Gene transcription counts were normalized by geometric mean of housekeeping genes before advanced analysis was performed with nSolver Version 4.0 (NanoString). RStudio (version 2022.07.2, build 576) was used with R version 4.2.1 for further statistical computing and graphic rendering. Partial least squares discriminant analysis (PLSDA) was performed with the ‘ropls’ package [33], followed by rendering of scatterplots with ggplot2 [34]. Pathway signature scores obtained from nSolver advanced analysis was used to summarize expression level changes of biologically related groups of genes. These pathway scores are derived from the first principal component analysis score for each sample based on its gene expression level for all genes measured within a specific pathway. Thus, a positive score would indicate that the pathway contains many upregulated genes, while a negative score would suggest the pathway had many downregulated genes. All heatmaps were created with the Complex Heatmap package [35].

### Flow cytometry analysis

Mice were euthanized at 19 months post-SCI and perfused with ice cold saline. An approximate 1-cm fragment of spinal cord tissue was dissected from the area surrounding the epicenter of the lesion site, washed again with saline to remove any residual or contaminant blood cells, and mechanically homogenized through a 70-μm filter followed by enzymatic digestion described in previous publications [36, 37]. For sham animals, a fragment of equal length was extracted from the corresponding site. Spinal cord tissue weights (mg) were recorded for each mouse immediately after harvest. For brain tissue processing, the left hemisphere was placed in complete Roswell Park Memorial Institute (RPMI) 1640 medium (Cat# 22400105, Invitrogen) and digested with the same method mentioned above.

Total cell counts were obtained after collecting all events for each spinal cord sample and were then normalized to mg tissue weight. All cells were blocked using TruStain FcX antibody (Clone: 93, Cat# 101320, Biolegend) prior to cell surface antibody staining. Leukocytes were identified after first gating for leukocyte scatter properties, singlets, and exclusion of the fixable viability dye Zombie Aqua (Cat# 423102, Biolegend). Living cells were identified as microglia (CD45^int^CD11b^+^) and infiltrating myeloid cells (CD45^hi^CD11b^+^) (CD45-eF450, 1:100, Cat# 48–0451-82, eBioscience; CD11b-APC-eF780, 1:100, Cat# 47–0112-82, eBioscience). Intracellular staining included TNF-PECy7 (1:50, Cat# 46–7114-82, Biolegend). Intracellular reactive oxygen species (ROS) was measured with staining dye H2DCFDA (DCF, 5 μM; Thermo-Fisher Scientific). Mitochondrial membrane potential was measured using MitoSpy Red CMXRos (Cat# 424802, Biolegend) according to manufacturer’s instructions. Data were acquired on a BD LSRFortessa cytometer using FACSDiva 6.0 (BD Biosciences) and analyzed using FlowJo (Treestar Inc). At least 5–10 million events were collected for each brain sample. Countbright™ Absolute Counting Beads (Invitrogen) were used to estimate brain cell counts per the manufacturer's instructions. Data were expressed as either cells/μl or total counts/hemisphere.

### Blood collection and processing

Blood collection and processing was performed as described previously [26]. Briefly, blood was collected from mice with a syringe through terminal cardiac puncture under isoflurane anesthesia and placed immediately into precoated EDTA tubes (Cat# 365974, BD Biosciences), which was gently inverted ten times for proper mixing. Blood was then centrifuged at room temperature (RT) on 500 × *g* for 15 min, 2500 × *g* for 10 min, and 2500 × *g* for 10 min to generate platelet-free plasma (PFP) [38]. Blood was kept at RT between collection and centrifugation for no more than 30 min to minimize the release of platelet EVs, which would occur under conditions such as cold temperature, agitation, and prolonged storage of blood. Afterwards, PFP was aliquoted into multiple tubes, flash frozen on dry ice, and stored at – 80 °C. At the start of further experiments, a single aliquot was thawed in the water bath at 37 °C to avoid multiple freeze/thaw cycles.

### Plasma and tissue EV isolation

EVs were isolated from PFP via ultracentrifugation protocol [26]. To isolate “total plasma EVs”, a 100-μL aliquot of PFP was diluted to 4 mL volume in filtered PBS (f-PBS), which was achieved with the use of a 0.22 μm PVDF filter (Cat# SLGV033RS, Millipore Sigma) prior to all EV isolation and characterization experiments. The diluted PFP was pipetted in a polypropylene tube (Cat# 326819, Beckman Coulter) and spun at 100,000×*g*, 4 °C for 90 min in a SW55Ti swinging bucket rotor in an Optima XE-90 Ultracentrifuge (Beckman Coulter). The resultant pellet was resuspended in 60 μL of f-PBS for downstream analysis by NTA and Western blot.

For EV isolation from frozen spinal cord and brain tissue, we used the same method described in our prior publication [26]. Tissue samples taken from – 80 °C storage were weighed and placed in a 50-mL conical tube containing a mixture of type III collagenase (Cat# LS004176; 40 U/mL; Worthington Biochemical) and Hibernate™-E medium (Cat# A1247601, Thermo-Fisher Scientific) at a ratio of 8 μL/mg tissue weight. Samples were placed on an orbital shaker at 37’C for dissociation. A cocktail mixture of protease (Cat#11697498001, Millipore Sigma) and phosphatase (Cat# 4906837001, Millipore Sigma) inhibitors was added to each sample at a volume equal to the enzyme solution. The sample was then serially centrifuged at 4 °C for 5 min at 300 × *g*, 10 min at 2000 × *g*, and 30 min at 10,000 × *g*, with the supernatant being completely transferred between steps. An equal volume of the remaining supernatant after the 10,000 × *g* spin was diluted with f-PBS to 4 mL in a thinwall, polypropylene tube and spun at 100,000 × *g* for 70 min at 4 °C with the SW55Ti. After removal of the supernatant, the tissue EV pellet was resuspended in 50 μL f-PBS.

### Nanoparticle tracking analysis and Western blot

Nanoparticle tracking analysis (NTA) was performed with ViewSizer® 3000 (HORIBA Scientific) as previously described [26]. In brief, EV samples isolated from plasma and tissue lysates were diluted with f-PBS to within the appropriate concentration of 1 × 10^7^ to 2 × 10^8^ particles/mL, which is the detectable range, and final EV concentration data are presented as “particles/mL”. Particle counts were integrated from 50 to 2000 nm in size, spanning the expected size range of various EV subtypes. For each sample, a minimum of 50 videos were recorded to generate enough particle counts (> 3000 particles) and accurate size distribution. Between each video, a magnetic stir bar was used to stir the samples automatically for 5 s in order to ensure proper mixing.

Western blot analysis was performed by loading equal volumes of resuspended EVs onto a 4–15% Criterion TGX Stain-Free Precast gels (Cat# 5678083, Bio-Rad) for SDS-PAGE and transferred onto nitrocellulose membranes (Cat#1704159, Bio-Rad). The membranes were blocked with 5% non-fat milk in PBST for an hour before being incubated in primary antibodies overnight. For detection of tetraspanin markers, the antibodies used were anti-CD63 (1:500, Cat# D263-3, MBL International) and anti-CD81 (1:1000, Cat# 10037S, Cell Signaling Technology). On the following day, the membrane was incubated for an hour in HRP-conjugated secondary antibodies and visualized with SuperSignal West Dura Extended Duration Substrate (Cat# 34076, Thermo-Fisher Scientific). The immunoblots were imaged with ChemiDoc TM MP system (Bio-Rad) and the optical density of the bands were quantified in the Image Lab program (Bio-Rad). Loading an equal volume of isolated EVs is a common practice for characterization of EVs markers with Western blot. According to the guidelines [39], no recommendations were made for selection of a housekeeping protein that is stably expressed in all EVs and can be used for Western blotting.

### Tissue Western blot analysis

The somatosensory cortex (tissue puncta) and the hippocampus were excised from the brain after killing and flash frozen in dry ice. Tissue samples were homogenized in RIPA lysis buffer (Cat# R0278, Sigma-Aldrich) supplemented with 1 × protease inhibitor cocktail (Cat# P8340, Sigma-Aldrich), Phosphatase Inhibitor Cocktail 2 (Cat# P5726, Sigma-Aldrich) and Phosphatase Inhibitor Cocktail 3 (Cat# P0044, Sigma-Aldrich), followed by sonication and centrifugation at 20,000 × *g* for 20 min. Pierce BCA method (Cat# 23227, Thermo-Fisher Scientific) was used to determine protein concentration. Samples were run on criterion TGX 4–20% gradient gels (Bio-Rad) for SDS-PAGE and transferred to 0.2 μm nitrocellulose membrane (Bio-Rad). Membranes were blocked with 5% non-fat skim milk in PBST and incubated overnight with primary antibodies diluted in blocking buffer at 4. The next day, membranes were incubated for 1 h in HRP-conjugated secondary antibodies. After the immunoblots were visualized with Supersignal West Pico PLUS Chemiluminescent Substrate (Cat# 34577, Thermo-Fisher Scientific), images were obtained with the ChemiDoc TM MP system (Bio-Rad). For quantification, optical density of signal bands was calculated with the Image Lab software (Bio-Rad). Primary antibodies and respective dilution rates are as followed: PSD95 (1:1000, Cat# 20665-1-AP, Proteintech), NCX1 (1:1000, Cat# 28447-1-AP, Proteintech), Nefl (1:1000, Cat# 2837S, Cell Signaling Technologies), PRAS40 (1:500, Cat# 21097-1-AP, Proteintech), GluN2B (1:500, Cat# 4212S, Cell Signaling Technologies), C1qA (1:1000, Cat# NBP1-51139, Novus Biologicals), PKCα (1:1000, Cat# 59754S, Cell Signaling Technologies), and β-actin (1:10,000; Cat# SAB1305567A1978, Sigma-Aldrich). All data are presented as the intensity of a target protein normalized by actin and compared to Sham/Male for each sample (expressed in fold of Sham/M).

### Fireplex microRNA assay and analysis

For analysis of miRNAs (miRs) cargo content within the isolated plasma and tissue EVs, we used the FirePlex miRNA Neurology V2 Panel (Cat# ab218371, Abcam). Total plasma and tissue lysate EVs were isolated by ultracentrifugation using the protocol described above and resuspended in 40 μL of f-PBS. Four groups were sent for analysis: Sham/Male, Sham/Female, SCI/Male, and SCI/Female. miRs were detected directly from these equal volume samples without the need for further processing and RNA isolation [40]. For data analysis, fluorescence intensity readout values were normalized using a geNorm-like algorithm in the Firefly Analysis Workbench [41]. Individual miR fluorescent intensity data were statistically analyzed and presented as linear fold change of the mean fluorescent intensity (MFI) data between relevant groups, with the adjusted (adj.) p-value being used for significance.

### Behavioral assessment

Basso mouse scale for locomotion: For assessment of hindlimb locomotion recovery, mice were placed in a flat, enclosed box of 62 × 42 cm dimensions and observed for at least 4 min by two trained observers blinded to the biological sex of the mice. Animals were rated using the Basso mouse scale (BMS) for locomotion on a scale of 0–9 based on joint movement, weight support, plantar stepping, and coordination of the hind limbs, with 0 being complete paralysis and 9 being normal locomotion [42]. Mice were tested for BMS scores on day 1, day 3 and weekly thereafter for up to 82 weeks post-injury.

Open field: Spontaneous motor activity was assessed with the open field (OF) test under dim light at 82 w post-injury. Each mouse was placed in a corner facing the wall of the open-field chamber (22.5 cm × 22.5 cm) and allowed to freely explore for 5 min. Parameters such as total distance travelled, average speed, time spent immobile, time freezing, and distance travelled in the inner zone were recorded with the Anymaze tracking program.

Y-maze: The Y-maze test was performed on week 82 post-injury to test the condition of spatial working memory in mice, as described previously [36, 43]. The Y-maze apparatus (Stoelting Co.) is consisted of three arms of identical length and width (A, B, C). During the test, one arm was selected randomly as the starting position for all mice and each mouse was placed in the maze to explore freely for 5 min. Arm entries were recorded, and an alternation was completed when the mouse enters three different arms consecutively. The percentage of alternation was calculated with the following equation: total alternations × 100/(total arm entries – 2). Mice that scored significantly higher than 50% (the chance level for choosing a unfamiliar arm) were considered to have functional spatial working memory.

Novel object recognition (NOR): NOR was performed at 82 w after SCI for assessment of non-spatial retention memory as described [36, 43]. After habituation in the test chamber on the first day, the amount of time mice spent with two identical objects were recorded on the second day. Afterwards, a novel object was introduced on the third day of testing and exploration time was recorded. Because mice inherently prefer to explore novel objects, a preference for the novel object (more time than chance [15s] spent with the novel object) indicates intact memory for the familiar object.

Novelty suppressed feeding (NSF): The conflict-based NSF test was performed at 82 weeks after SCI to assess depressive-like behavior as described in previous studies [16, 44]. The latency time for a mouse to reach the food pellets in the center of a brightly lit, novel open-field arena was recorded within a maximum time of 10 min. For animals that did not eat the food pellet within the maximum allowed time, a latency of 600 s was assigned to them. To control for differences in hunger or motivation, the latency time to eat was also recorded upon returning each mouse to their respective home cage [16, 44].

### Statistical analysis

All quantitative data are presented as individual data points with mean ± SEM. All statistical analysis was performed on Graphpad Prism Version 9.5.0 for Windows (Graphpad Software, RRID: SCR_002798). Normal distribution of data was assessed with the Shapiro–Wilk test. For multiple comparisons, one-way or two-way ANOVA were performed followed by Tukey’s multiple comparisons post hoc test for parametric (normality and equal variance passed) data. For NTA and WB analysis of EV samples, Mann Whitney U test was performed between two groups. For analysis of miR assay data, two-way ANOVA test was used and followed by Tukey’s multiple comparisons test to compare main effects of biological sex or injury. Kaplan–Meier survival curves were analyzed using the log-rank Mantel–Cox test. BMS scores were analyzed with two-way ANOVA for repeated measurements followed by Sidak’s multiple comparisons post hoc test. Statistical analysis in each assay is detailed in figure legends and a p-value ≤ 0.05 was considered statistically significant.

## Results

### SCI leads to sexually dimorphic transcriptional responses in the aged brain

**Fig. 1:**
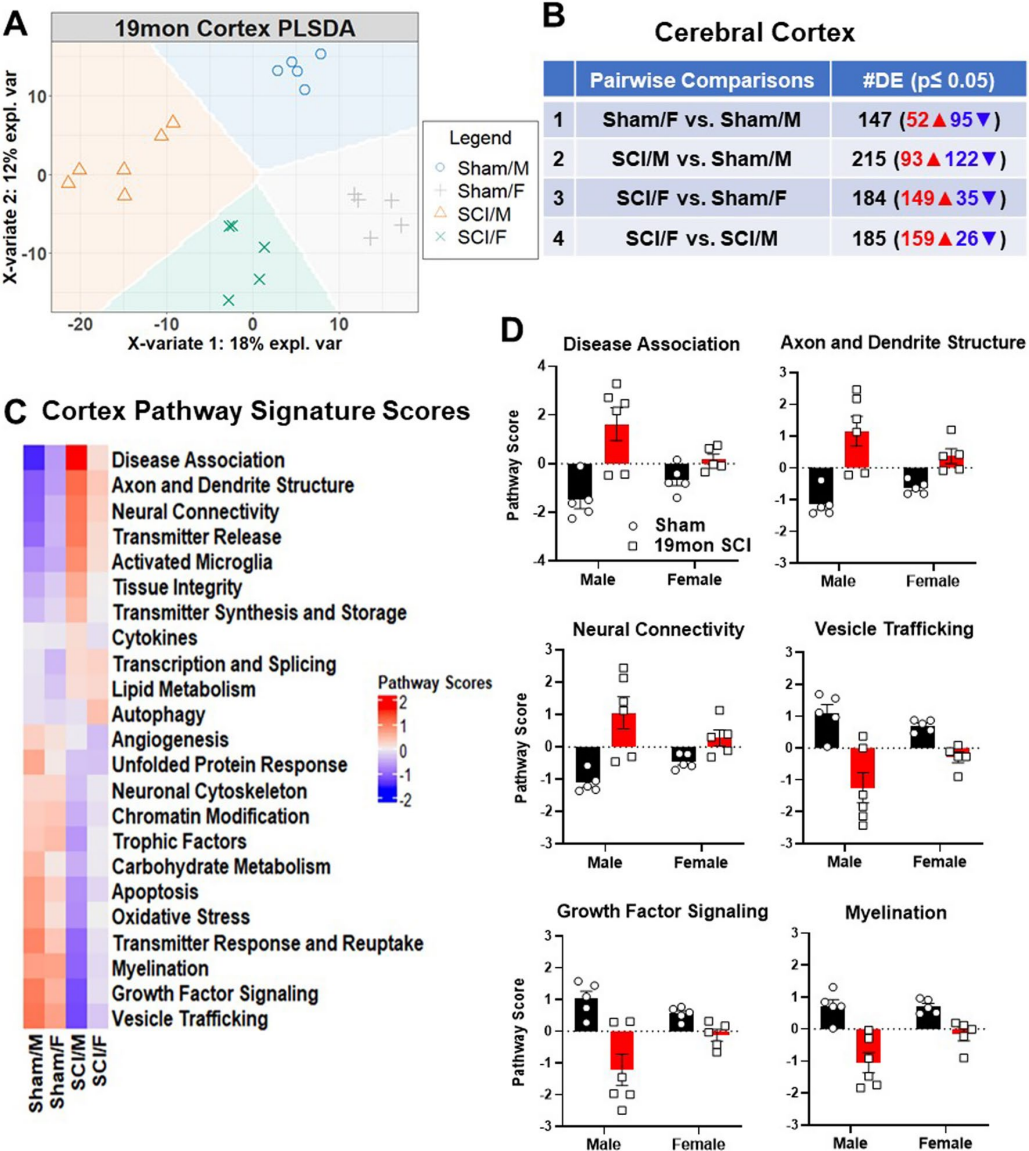
Chronic SCI alters the transcriptome in the cerebral cortex. Age-matched male and female C57BL/6 mice were subjected to moderate SCI at 10–12 weeks old. NanoString Neuropathology panel was used to assess transcriptional changes in the somatosensory cortex at 19 months (mon) post-injury. **A** Partial least square differentiation analysis (PLSDA) was performed with all normalized gene counts from the four sample groups and displayed clear clustering along the x- and y-axis which showed the degree of variation explained. **B** Pairwise comparison and number of differentially expressed genes (DEGs, unadjusted *p* < 0.05) in each comparison set. **C** Heatmap of pathway signature scores for DEGs showed gene groups that are either predominantly upregulated or downregulated by sex and SCI. **D** Bar graph of pathway signature scores for top 3 downregulated and upregulated pathways in SCI/M group compared to Sham/M. *n* = 5–6 mice/group

To explore how SCI affects the aging brain and the role that biological sex plays in transcriptional activation in the brain, we subjected age-matched male and female young adult C57BL/6 mice to a moderate contusion injury. Using the NanoString Neuropathology panel, we examined transcriptional changes for 770 genes related to neurodegeneration and pathology in cerebral cortex (*n* = 5–6 mice/group) and hippocampus tissue (*n* = 5–6 mice/group) at 19 months (mon) after SCI. PLSDA was used to differentiate samples on normalized transcriptional counts of all tested genes in the assay panel. Gene expression data of the cortex effectively separated the four groups along the two main components of sex and injury, with injury driving up to 18% of the observed variation and sex contributing to a slightly lesser degree at 12% (Fig. 1A). For pairwise comparison, we performed group by group analysis in the following order: (1) Sham/F vs. Sham/M; (2) SCI/M vs. Sham/M; (3) SCI/F vs. Sham/F; (4) SCI/F vs. SCI/M. Among the four pairwise comparison sets, there were 147 differentially expressed genes (DEGs) (52 upregulated, 95 downregulated) for Sham/F vs. Sham/M, 215 DEGs (93 upregulated, 122 downregulated) for SCI/M vs. Sham/M (Male injury genes), 184 DEGs (149 upregulated, 35 downregulated) for SCI/F vs. Sham/F (Female injury) and 185 DEGs (159 upregulated, 26 downregulated) for SCI/F vs. SCI/M (Fig. 1B). Overall, male mice predominately showed gene downregulation, whereas females showed greater upregulation after SCI.

**Fig. 2:**
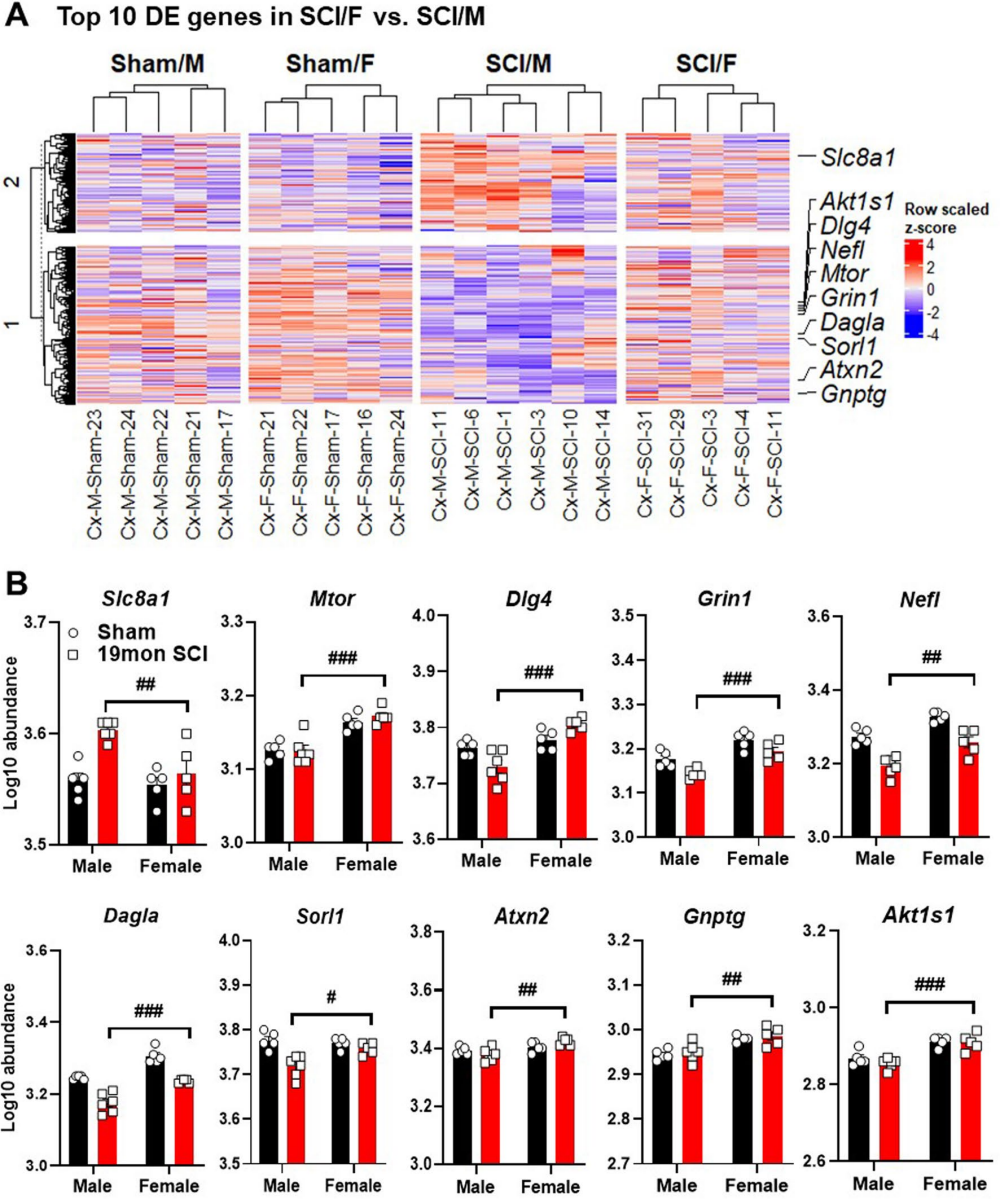
Top 10 genes modified by biological sex and SCI in the somatosensory cortex. **A** Heatmap of all normalized genes and samples with gene annotations for top 10 genes. Gene count for each sample were converted to z-score and all samples within each group are clustered by k-means. **B** Log10 abundance of the top 10 genes modified by biological sex. *n* = 5–6 mice/ group. ^#^*p* < 0.05, ^##^*p* < 0.01, ^###^*p* < 0.001 vs. SCI/M group. Two-way ANOVA followed by Tukey’s multiple comparisons test

Through heatmap clustering of the average scores for each group, we identified disease association, axon and dendrite structure, and neural connectivity as the top three upregulated pathways in the cerebral cortex between male and female SCI mice (Fig. 1C). In general, the signature score of the top three upregulated pathways showed higher elevation in SCI/M group, suggesting that specific pathological changes might be more prominent in male mice after injury (Fig. 1D). On the other hand, vesicle trafficking, growth factor signaling, and myelination were the top three pathways in which SCI/M showed overall lower scores compared to SCI/F mice (Fig. 1C, D Additional file 1: Fig. S1A, B). Further exploration of the DEGs based on relative fold change between SCI/F and SCI/M showed that the top 10 most modified genes are predominantly expressed at higher levels in female mice, except for two genes (Fig. 2A, B). Out of these 10 genes: 6 genes (*Slc8a1, Mtor, Dlg4, Grin1, Nefl, Dagla*) are related to axon and dendrite structure, 2 genes (*Sorl1, Atxn2*) are in the disease association pathway, 1 gene (*Gnptg*) is a marker of activated microglia, while *Akt1s1* regulates carbohydrate metabolism.

**Fig. 3:**
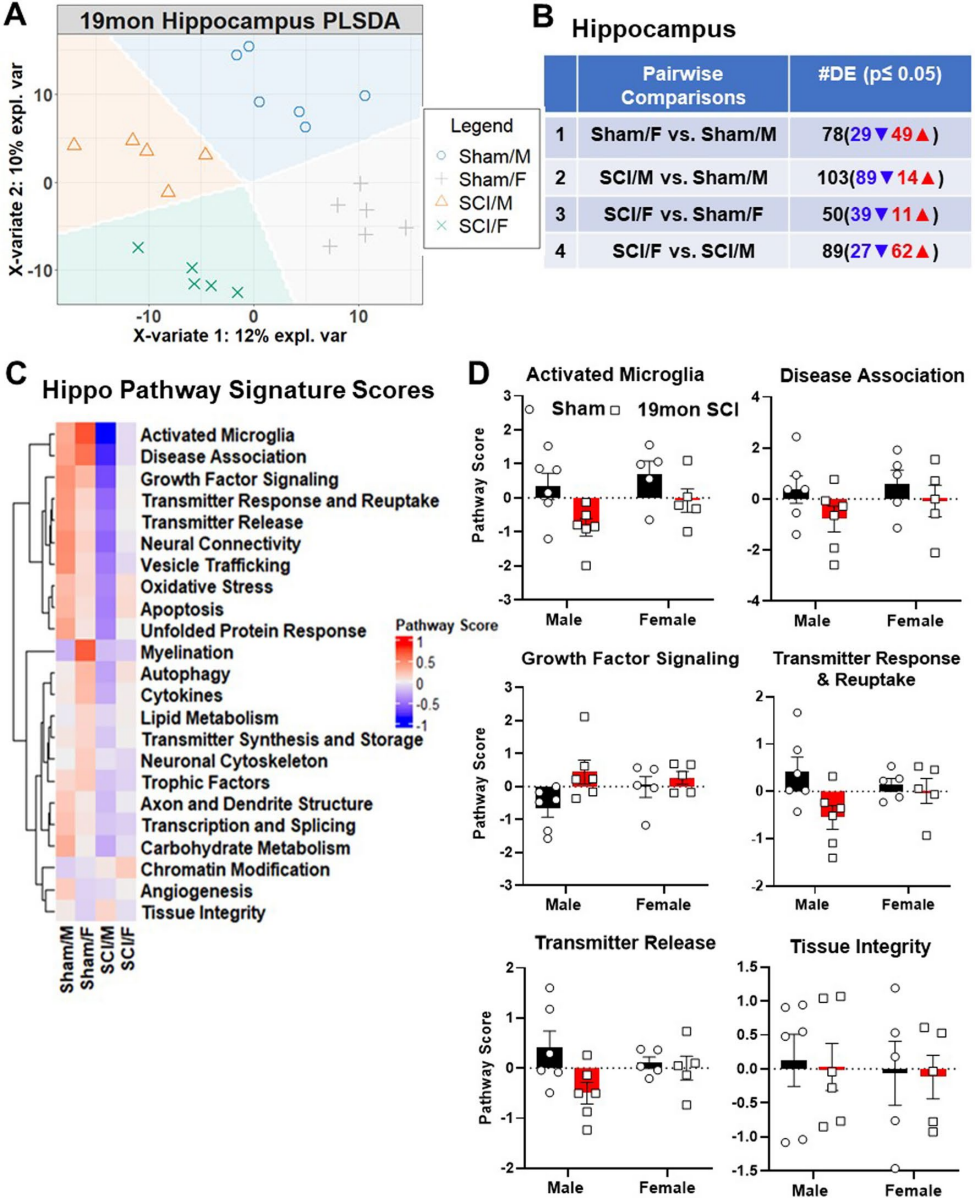
Long-term SCI alters transcription of genes related to neuropathology in the hippocampus. **A** PLSDA of normalized gene counts revealed distinct clustering by injury and biological sex. **B** Number of DEGs (p-value < 0.05) in each set of pairwise comparison. **C** Heatmap of pathway signature scores for DEGs in the hippocampus. **D** Bar graph of pathway signature scores for top 5 pathways downregulated by SCI in males and the top upregulated pathway in SCI/M group. *n* = 5–6 mice/group

Due to its importance in controlling learning and memory and its known susceptibility to pathological changes induced by aging and stress [45, 46], we next examined the hippocampus for transcriptional changes. PLSDA of the hippocampus samples showed similar patterns as the cortex with clear separation of the four groups by sex and injury (Fig. 3A). However, unlike in the cerebral cortex, injury only contributed to 10% of the variation between groups, whereas biological sex seems to be the main driver for cluster differences at 12%. Consistent with this, less DEGs were found out of the four pairwise comparison sets (Fig. 3B).

**Fig. 4:**
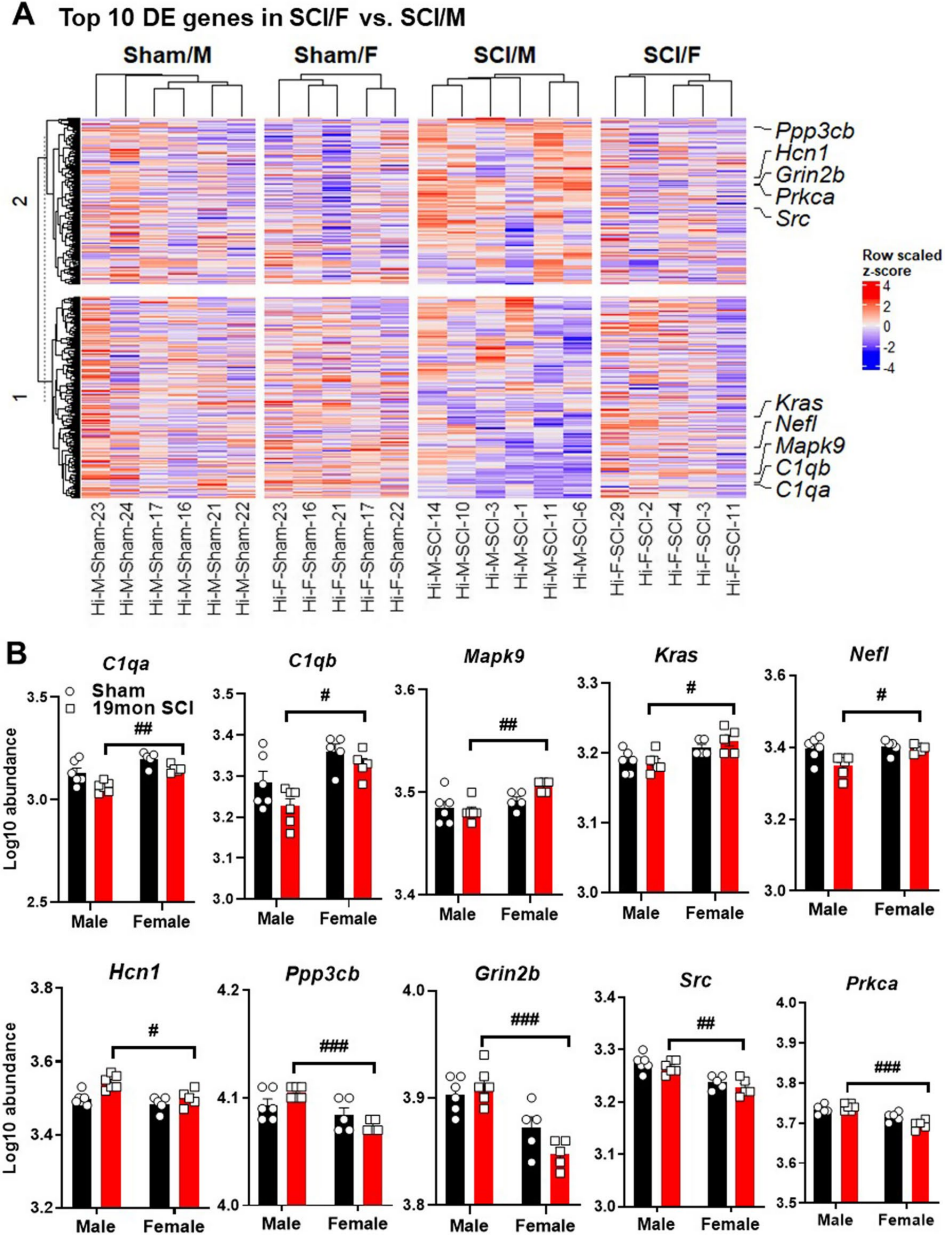
Top 10 genes modified by biological sex in the hippocampus. **A** Heatmap of all normalized genes and samples with annotations for top 10 genes. Gene count for each sample were converted to z-score and k-means clustering was used within group. **B** Normalized transcription count of the top 10 genes presented as Log10 abundance. n = 5 ~ 6 per group. ^#^*p* < 0.05, ^##^*p* < 0.01, ^###^*p* < 0.001 vs. SCI/M group. Two-way ANOVA followed by Tukey’s multiple comparisons test

An overview of the number of DEG pathway signature scores for the hippocampus identified activated microglia, disease association, growth factor signaling, transmitter response and reuptake along with transmitter release as the main pathways of change (Fig. 3C, D). The predominant trend in these pathways was lower scores for SCI/M group compared to SCI/F. The only pathway that had a higher score in SCI/M mice, as demonstrated in the heatmap clustering, was tissue integrity. Isolation of male-specific injury genes and female specific injury genes showed robust differences in the expression level and pathways activated by chronic SCI. Male injury genes showed far more DEGs in the pathways regulating activated microglia, disease association and growth factor signaling (Additional file 1: Fig. S2A, B). Furthermore, the top 10 DEGs in a group comparison between SCI/F vs. SCI/M showed marked differences across sexes (Fig. 4A, B). Within these 10 genes, 2 regulated functions pertaining to activated microglia (*C1qa, C1qb)*. Two genes related to apoptosis (*Mapk9, Kras*); *Nefl* and *Hcn1* were involved with regulation of the axon and dendrite structure, with another 2 genes (*Ppp3cb, Grin2b*) mainly regulating processes of disease association and both *Src* and *Gabrb* involved with neural connectivity. Collectively, our results show sexual and regional dimorphism of brain gene expression during aging following long-term SCI.

**Fig. 5:**
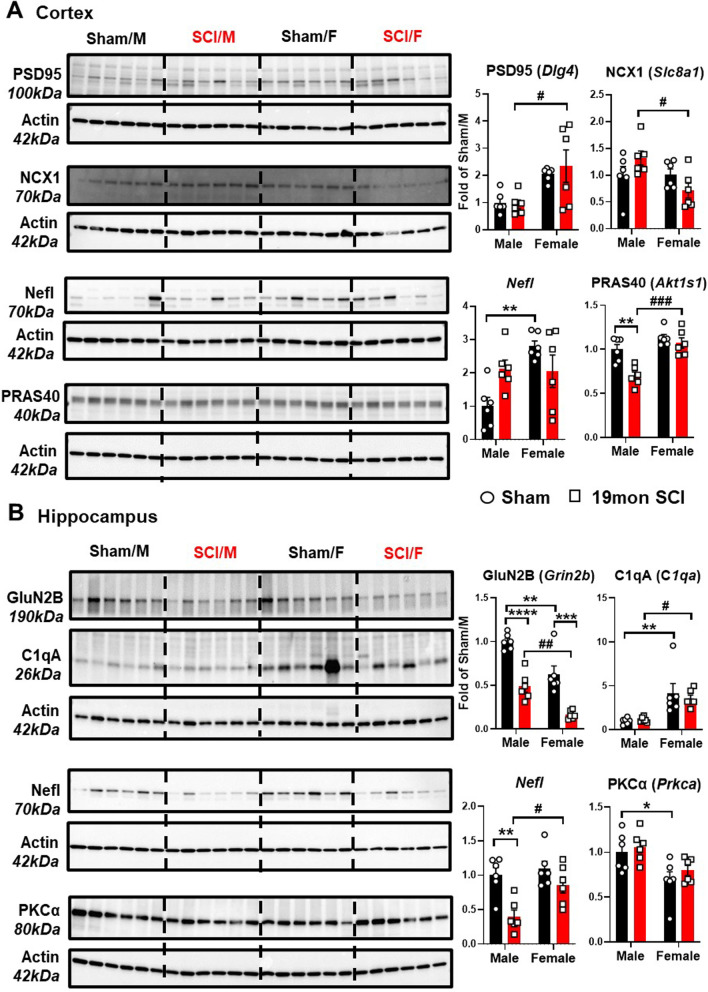
Validation of Nanostring DEGs with Western blot. Tissue samples from the cortex (**A)** and hippocampus (**B)** were processed for Western blot analysis to validate the top DEGs derived from Nanostring. **A** Expression of the DEG markers PSD95 (Dlg4), NCX1 (Slc8a1), Nefl (Neurofilament Light), and PRAS40 (Akt1s1) in cortex tissue. **B** Expression of the DEG markers GluN2B (Grin2b), C1qA (C1qa), Nefl, and PKCα (Prkca) in the hippocampus. *n* = 6 mice/group. *p < 0.05, **p < 0.01, ***p < 0.001, ****p < 0.0001 vs. Sham groups, ^#^*p* < 0.05, ###p < 0.001 vs. SCI/Male. Two-way ANOVA followed by Tukey’s multiple comparison

For validation of the DEGs, we used Western blot analysis to examine the protein expression levels of the most significant DEG in each region (Fig. 5, *n* = 6 mice/group). Significant differences between SCI/M and SCI/F were observed in the proteins regulated by PSD95 (*Dlg4*), NCX1 (*Slc8a1*), and PRAS40 (*Akt1s1*), which followed the same trend seen in NanoString analysis of the somatosensory cortex (Fig. 5A and Additional file 1: Fig. S3A). Although Nefl (Neurofilament light) did not show significant difference between SCI groups in the cortex, there was a baseline difference between Sham/M and Sham/F mice. In the hippocampus, Nefl was significantly higher in the SCI/F mice compared to SCI/M (Fig. 5B and Additional file 1: Fig. S3B). The protein expression level of GluN2B (*Grin2b*) and C1qA (*C1qa*) also followed the same trend as NanoString. The expression level of (PKCα) (*Prkca*) was significantly reduced in Sham/F compared to Sham/M, but no significant sex difference was observed in SCI groups.

### Chronic SCI alters cellular function in both male and female

**Fig. 6:**
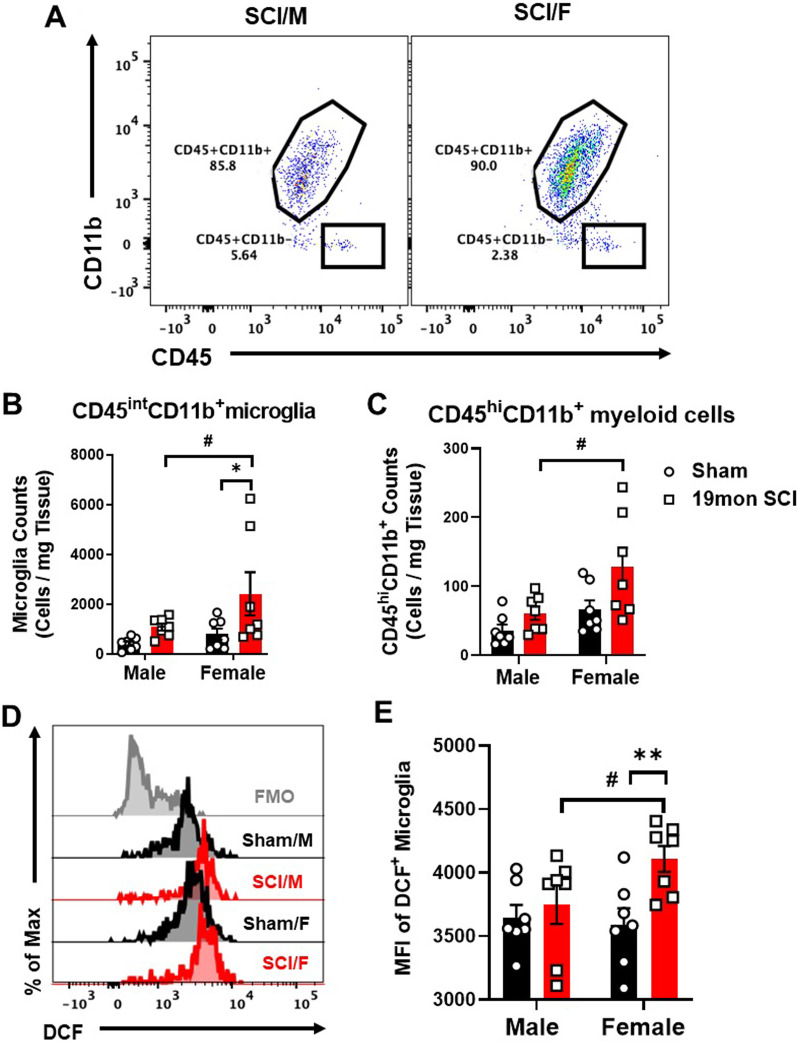
Chronic SCI alters cellular inflammation in the spinal cord of male and female. **A** Representative dot plots depict the relative immune cell composition in the lesion area of the spinal cord at 19 months (mon) post-injury. **B**, **C** Cell counts normalized by tissue weight showed increased number of CD45^int^CD11b^+^ microglia (**B)** and CD45^hi^CD11b^−^ infiltrating myeloid cells (**C)** in the spinal cord of SCI/F mice. **D** Representative histogram showed the relative production of ROS in microglia as measured by H2DCFDA (DCF) dye. **E** Mean fluorescence intensity (MFI) showed higher ROS production levels in SCI/F compared to Sham/F and SCI/M groups. n = 7 per group. *p < 0.05, **p < 0.01 vs. Sham group; #p < 0.05 vs. SCI group. Two-way ANOVA followed by Fisher’s LSD test for multiple comparison (**A**, **E**) and by Tukey’s multiple comparisons test (**B**)

To evaluate neuroinflammation and leukocyte function in the spinal cord after SCI, we performed flow cytometry on extracted segments of the injury site and a segment of equal length in sham groups (Additional file 1: Fig. S4 for gating strategy). At 19mon post-injury, no statistically significant difference in microglia number was observed between SCI/M and Sham/M mice, but female mice showed significantly higher microglia counts in the SCI group compared to Sham/F (Fig. 6A, B, n= 7/group). SCI-induced increases in peripherally derived CD45^hi^CD11b^+^ myeloid cells were also blunted in male mice, whereas SCI/F showed significantly higher cell counts than SCI/M (Fig. 6C). Next, we evaluated ROS production in microglia. No sex differences were observed in the overall percentage of DCF + microglia (Fig. 6D), however the mean fluorescence intensity (MFI) was significantly higher in SCI/F mice compared to their injured male counterparts (Fig. 6E). These results suggest a more marked inflammatory response in female mice after 19mon SCI, while male mice show little changes.

**Fig. 7:**
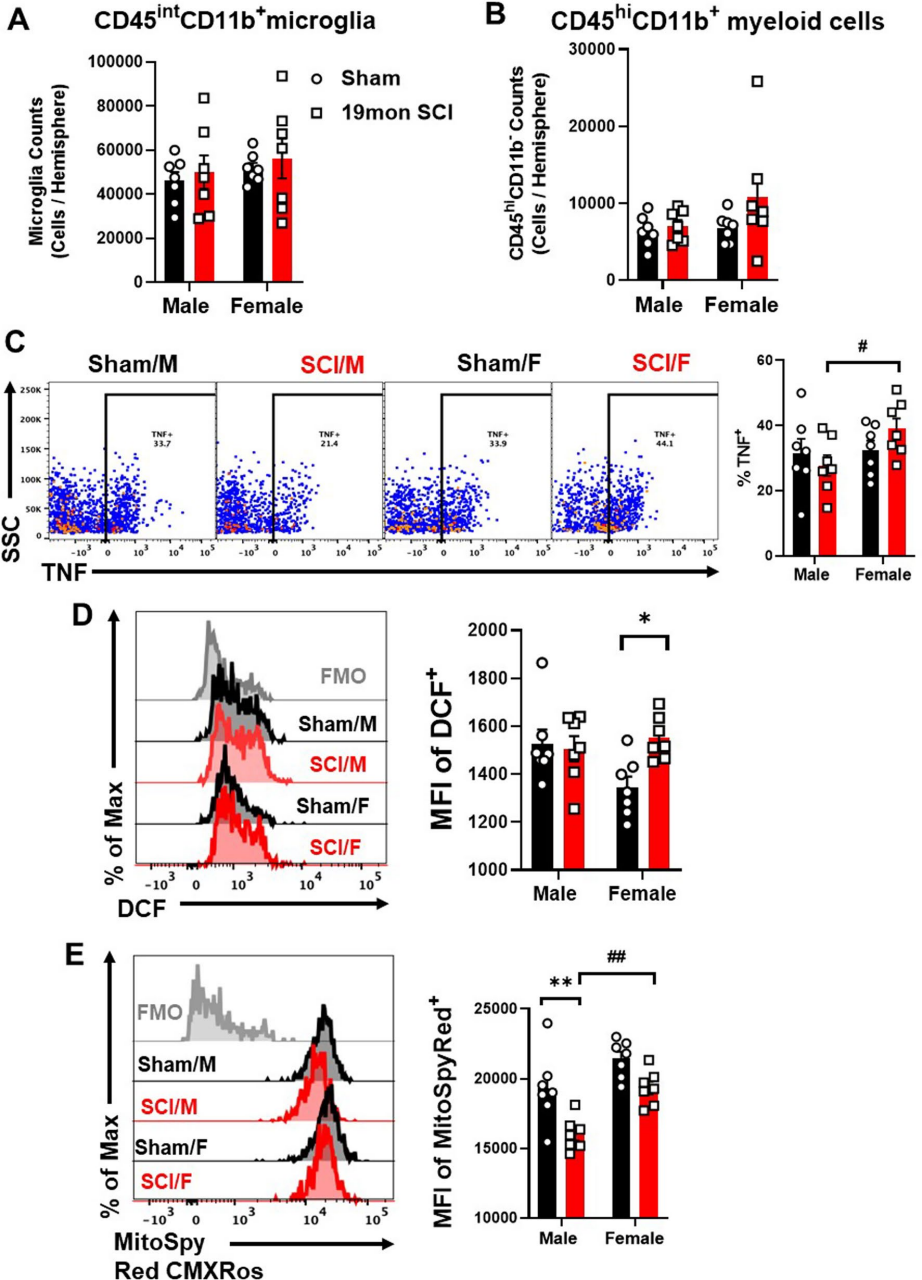
Neuroinflammation in the brain can be detected long after initial SCI. **A**, **B** Flow cytometry quantified the cell counts of CD45^int^CD11b^+^ microglia (**A)** and infiltrating CD45^hi^CD11b^−^ myeloid cells (**B**). **C** Representative dot plots and percentage of TNF + cells in the microglia. **D** Representative histogram and MFI of H2DCFDA (DCF) showed increased levels of ROS production in the brain of SCI/F group compared to Sham/F. **E** Representative histogram and MFI of MitoSpy Red CMXRos showed decreased mitochondrial activity in SCI/M compared to Sham/M while SCI/F showed no difference compared to Sham/F and significantly higher than SCI/M. n = 7 per group. *p < 0.05, **p < 0.01 vs. Sham group; ^#^*p* < 0.05, ^##^*p* < 0.01 vs. SCI group. Two-way ANOVA followed by Tukey’s multiple comparisons test

In the brain, there were no injury or sex differences in microglia or CD45^hi^ myeloid cell counts (Fig. 7A, B, n= 7/group). Remarkably, the proinflammatory cytokine TNF and ROS production were produced more abundantly in SCI/F microglia compared to SCI/M (Fig. 7C, D). Decreased mitochondrial activity in microglia was present in male mice but not female mice (Fig. 7E).

### Certain EV subtypes are more elevated in males after chronic SCI

**Fig. 8:**
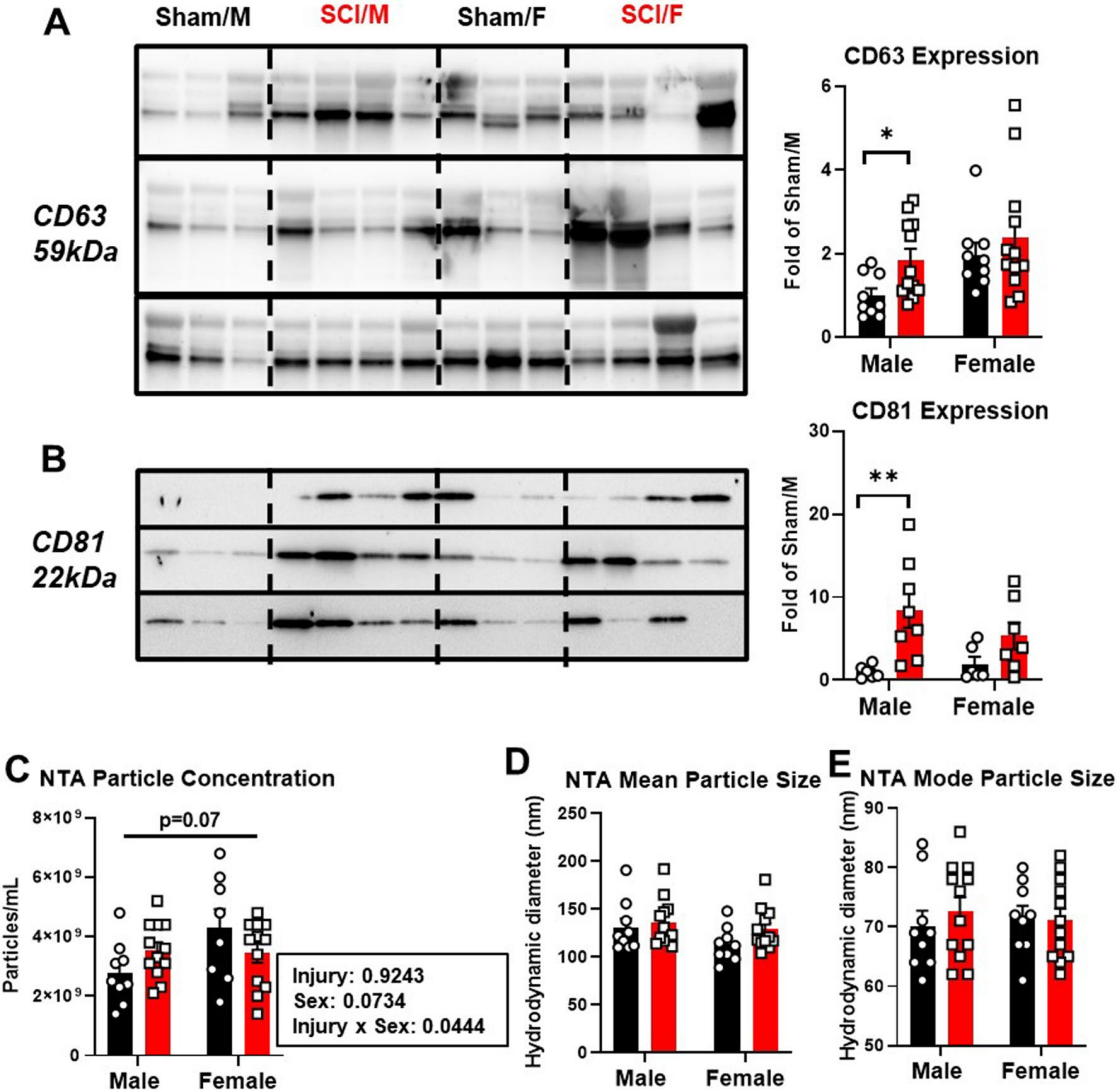
Plasma EV subtypes are increased robustly in males at 19 months post-injury. **A**, **B** Western blot was performed on total plasma EVs for tetraspanin EV markers CD63 (**A)** and CD81 (**B)** with quantification of densitometry showing robust increase in SCI/M compared to Sham/M. **C**–**E** Nanoparticle Tracking Analysis (NTA) was performed on the same samples with particle concentration (**C)** and mean/mode particle size (**D**, **E)** shown. *n* = 9 for sham groups and 12 for SCI groups. *p < 0.05, **p < 0.01 vs. Sham group. Multiple Mann–Whitney *U* tests for pairwise comparison

We recently reported that SCI modifies circulatory EV counts and content in the blood plasma at the acute phase of injury [26]. To continue this line of investigation, we sought to determine whether the same changes could be observed in chronic SCI. Using EV samples isolated from total plasma (*n* = 9 for sham groups, *n* = 12 for SCI groups), we performed Western blot analysis to examine the protein expression levels of tetraspanin EV markers CD63 and CD81. Of the two markers tested, males had a more robust increase than females with injury (Fig. 8A, B and Additional file 1: Fig. S5). Quantification of particle concentration and size distribution by NTA with the Viewsizer 3000 showed significant interaction between biological sex and injury (*n* = 9 for sham groups, *n* = 12 for SCI groups, *F*_(1,37)_ = 4.330, *p* = 0.0444) at 19mon post-injury (Fig. 8C–E and Additional file 1: Fig. S5).

**Fig. 9:**
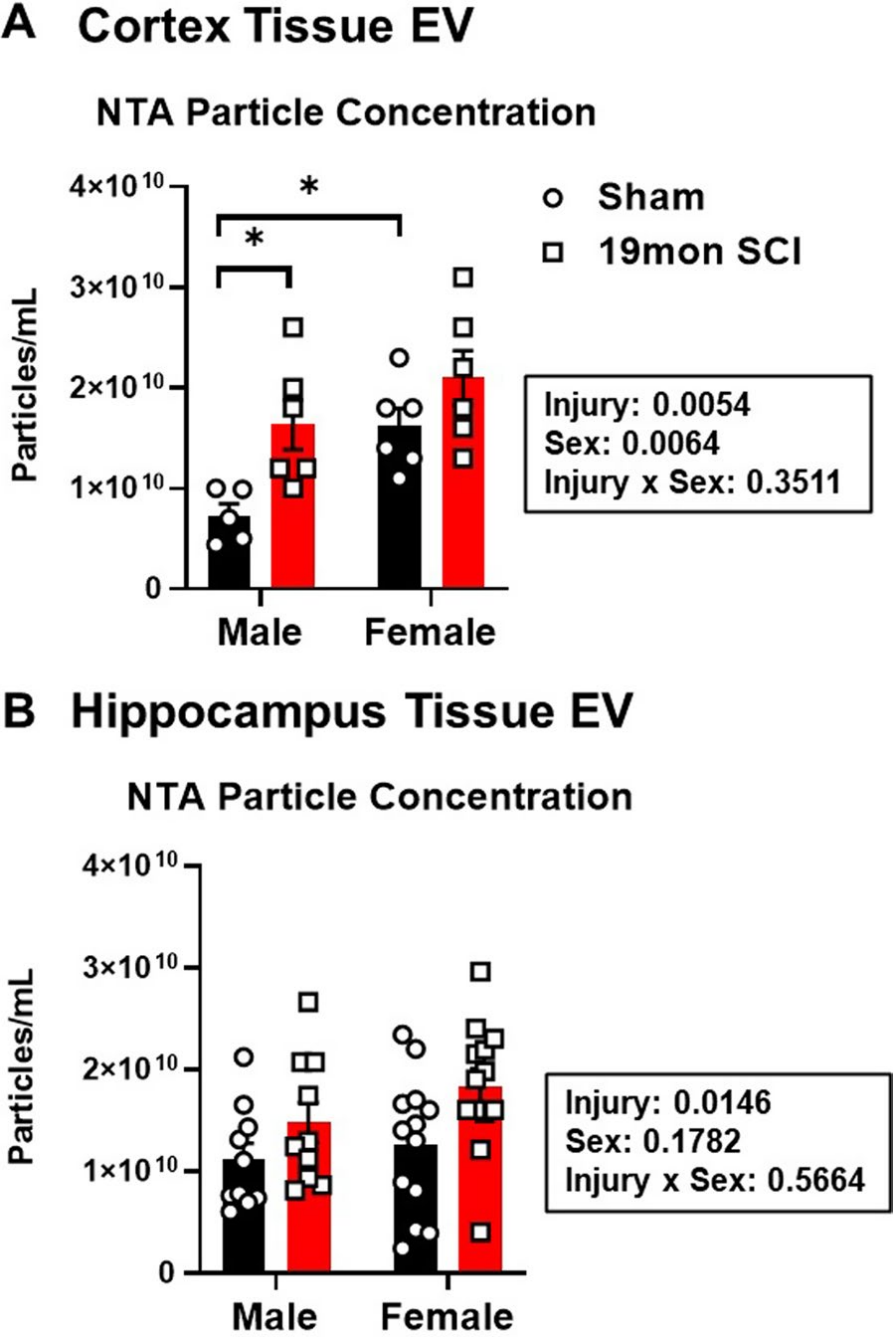
SCI increases EV particle counts in the brain. Particle concentration was examined by Nanoparticle Tracking Analysis (NTA) of EVs isolated from the somatosensory cortex (*n* = 5–6/group) (**A**) and hippocampus regions (*n* = 10 for males, *n* = 13 for female groups) (**B**). *p < 0.05 vs. Sham/M group. Two-way ANOVA followed by Tukey’s multiple comparisons test

Assessment of particle counts in EV samples isolated from the somatosensory cortex showed significant main effects for both sex (*n* = 6/group, F_(1,19)_ = 9.365, p = 0.0064) and injury (*F*_(1,19)_ = 9.830, *p* = 0.0054) at 19mon after SCI; post hoc multiple comparisons found significantly higher particle concentration levels in SCI/M (*p* = 0.0277) and Sham/F (*p* = 0.0308) compared to Sham/M (Fig. 9A). In the hippocampus, particle counts showed a non-statistical trend for increase between sham and injury groups for each sex, along with a significant main effect of injury (*n* = 10 for male groups, *n* = 13 for female groups, *p* = 0.0146, Fig. 9B). However, post hoc pairwise analysis of the four groups showed no significant difference between SCI/M and SCI/F groups.

### Long-term SCI alters plasma and tissue EVs microRNA content

We have shown that SCI alters the brain transcriptome along with EV parameters in the plasma and brain [26]. Based on these earlier findings, we hypothesized that changes to bioactive cargo in circulating EVs, such as miRs, may contribute to the neuropathology. miRs are powerful transcriptional regulators that target multiple mRNAs based on complementary base-pairing. By utilizing the Fireplex Neurology assay, we were able to compare the miR content of EVs isolated from four different compartments: plasma (*n* = 6/group), spinal cord tissue (lesion area, *n* = 6/group), motor/somatosensory cortex (*n* = 7/group), and hippocampus (*n* = 7/group). Of the more than 60 miRs tested, we found two miRs in the spinal cord, the cortex, and the hippocampus, separately that had statistically significant interaction effects between injury and sex (Table 1). In spinal cord EVs, *miR-301a-3p* increased with injury in males but decreased in females, whereas the opposite trend can be seen with *miR-98-5p* (Additional file 1: Fig. S6A). In the somatosensory cortex, the two miRs with statistically significant interaction effects (*miR-382-5p* and *miR-151a-3p*) increased in males after injury but remained unchanged between Sham/F and SCI/F (Additional file 1: Fig. S6B). In the hippocampus, *miR-451a* levels were highest in Sham/F mice and decreased after chronic SCI, while both Sham/M and SCI/M groups showed relatively lower expression levels (Additional file 1: Fig. S6C). The other miR that showed significant interaction effects in the hippocampus was *miR-181b-5p*, which showed lower levels in SCI/M compared to Sham/M and increased expression in SCI/F compared to Sham/F.

**Table 1:** miRNA cargo of tissue derived EVs are affected by sex and injury interaction

Probes	▲ OR▼ Linear MFI fold change			
	SCI vs. Sham		Female vs. Male	
*Spinal cord*	Male	Female	Sham	SCI
hsa-miR-301a-3p	2.21	**0.56**	2.06	**0.52**
hsa-miR-98-5p	**0.81**	1.85	**0.99**	2.26
*Cortex*				
hsa-mir-382-5p	1.40	**0.96**	1.11	**0.76**
hsa-mir-151a-3p	2.43	**0.92**	1.45	**0.55**
*Hippocampus*				
hsa-miR-451a	**0.95**	**0.12**	4.28	**0.53**
hsa-miR-181b-5p	**0.85**	1.21	**0.86**	1.22

Linear fold change of the mean fluorescent intensity (MFI) data between relevant groups are shown, with either Sham or Male as the reference groups. n = 6/group for spinal cord EVs; n = 7/group for cortex and hippocampus EVs

We next analyzed miR levels based on main effect factors, which yielded more differential expressed miRs with an adjusted p-value of less than 0.05. By examining the main effects of biological sex, we found seven miRs in EVs derived from hippocampus tissue, seven from spinal cord and two miRs in plasma (Table 2). The hippocampal EVs had five miRs (*miR-21-5p, miR-7-5p, let-7f-5p, miR-338-3p, miR-146a-5p*) that showed a significant increase in female mice relative to male mice. On the other hand, the *miR-124-3p* and *miR-34a-5p*, showed significantly lower levels in female mice. Furthermore, in the spinal cord tissue derived EVs, we found seven miRs (*let-7f-5p, miR-195-5p, miR-370-3p, miR-98-5p, let-7g-5p, miR-17-5p, let-7b-5p*) were elevated in female mice compared to males. In circulating EVs, we found two miRs that showed significant sex effects, with *miR-214-3p* showing significant increase of up to 1.76-fold change in females compared to males. Lastly, *miR-103a-5p* showed significant decrease in circulating plasma EVs. Taken together, we found that EVs in the hippocampus and spinal cord largely showed increased levels of specific neurology-related miRNA cargo in females compared to males.

**Table 2:** Tissue and circulating EV miR cargo is modified by biological sex

Probes	▲ OR▼	Linear MFI fold change	adj. *p*-value
*Hippocampus*			
hsa-miR-21-5p	▲	3.52	*
hsa-miR-7-5p	▲	2.34	*
hsa-let-7f-5p	▲	2.06	*
hsa-miR-338-3p	▲	1.70	*
hsa-miR-146a-5p	▲	1.58	*
hsa-miR-124-3p	▼	**0.69**	*
hsa-miR-34a-5p	▼	**0.63**	**
*Spinal cord*			
hsa-let-7f-5p	▲	2.06	**
hsa-miR-195-5p	▲	1.80	*
hsa-miR-370-3p	▲	1.64	*
hsa-miR-98-5p	▲	1.56	*
hsa-let-7g-5p	▲	1.46	*
hsa-miR-17-5p	▲	1.39	*
hsa-let-7b-5p	▲	1.34	*
*Plasma*			
hsa-miR-214-3p	▲	1.76	*
hsa-miR-103a-3p	▼	**0.82**	*

Linear fold change of the mean fluorescent intensity (MFI) data between groups are shown as well as adjusted (adj.) p-value of significance. For all data, male is used as the reference group. *n* = 6/group for plasma and spinal cord EVs, *n* = 7/group for cortex and hippocampus. *p < 0.05, **p < 0.01 vs male group. Two-way ANOVA following Tukey’s multiple comparisons test

In contrast, injury itself had a more significant impact on EV miR content (Table 3). Greater changes were observed in the cargo content of EVs derived from the hippocampus than the cortex and spinal cord. In the hippocampus region, where the most changes were observed, *let-7b-5p* and three other miRs (*miR-24-3p, miR-328-3p, miR-370-3p*) showed marked increase in SCI groups, whereas five miRs were decreased (*let-7i-5p, miR-103a-3p, miR-107, miR-22, miR-532-5p*). This is contrasted by the injury-induced differential expression in cortex EVs, which only showed three miRs (*miR-323a-3p, let-7d-5p and miR-128-3*), all of which were upregulated in injury groups compared to sham. In the spinal cord, *miR-497-5p and let-7b-5p* showed significant increase at 19mon post-SCI. A total of five plasma-derived EV-encapsulated miRs showed significant injury-dependent changes, the majority of which were downregulated in SCI groups. Collectively, these data indicate that biological sex influences the release of EVs and cargo shuttling in chronic SCI.

**Table 3:** Tissue and circulating EV miR cargo is modified by chronic SCI

Probes	▲ OR▼	Linear MFI Fold Change	adj. p-value
*Hippocampus*			
hsa-miR-370-3p	▲	1.89	*
hsa-let-7b-5p	▲	1.55	*
hsa-miR-328-3p	▲	1.36	*
hsa-miR-24-3p	▲	1.30	*
hsa-miR-103a-3p	▼	**0.74**	*
hsa-miR-107	▼	**0.73**	*
hsa-miR-532-5p	▼	**0.7**	*
hsa-let-7i-5p	▼	**0.69**	*
hsa-miR-22-3p	▼	**0.64**	*
*Cortex*			
hsa-miR-323a-3p	▲	1.62	*
hsa-let-7d-5p	▲	1.21	*
hsa-miR-128-3p	▲	1.16	*
*Spinal cord*			
hsa-mir-214-3p	▲	2.09	*
hsa-miR-497-5p	▲	1.63	*
hsa-let-7b-5p	▲	1.33	*
hsa-mir-103a-3p	▼	**0.89**	*
*Plasma*			
hsa-let-7d-5p	▲	1.08	*
hsa-miR-15a-5p	▼	**0.82**	*
hsa-miR-93-5p	▼	**0.79**	*
hsa-miR-16-5p	▼	**0.76**	*
hsa-miR-486-5p	▼	**0.68**	*
hsa-miR-451a	▼	**0.65**	*

Linear fold change of the mean fluorescent intensity (MFI) data are shown as well as adjusted (adj.) p-value of significance. For all data, sham is used as the reference group. *n* = 6/group for plasma and spinal cord EVs, *n* = 7/group for cortex and hippocampus. *p < 0.05 vs Sham group. Two-way ANOVA following Tukey’s multiple comparisons test

### Male and female mice exhibit similar mortality and neurological functional impairment after chronic SCI

**Fig. 10:**
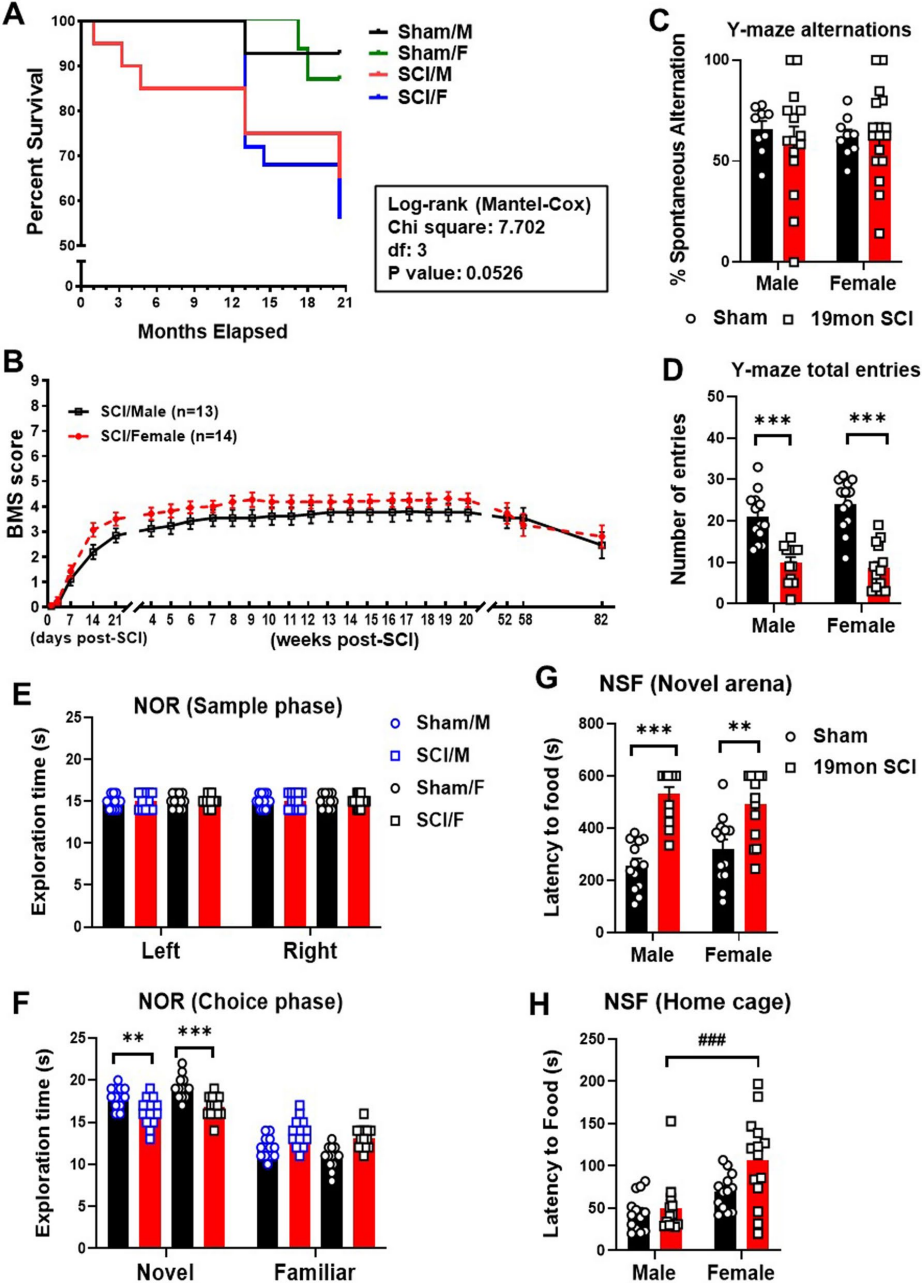
SCI mice in both male and female display neurological dysfunction at 19 months post-injury.** A** Survival plot of male and female mice in months elapsed for study. Compared to sham mice for either sex, SCI injury groups of both sexes showed decreased survival rates, with no difference in survival between biological sex. Kaplan–Meier survival curves were analyzed using the log-rank Mantel–Cox test. **B** BMS scores were recorded weekly to quantify hindlimb locomotor recovery after SCI. **C**, **D** Spontaneous alterations in Y-maze showed no injury or sex differences between groups, but total arm entries showed significant decrease in SCI groups of both sexes. **E**, **F** Novel object recognition (NOR) test showed significant decrease in exploration time of the novel object in both sexes after chronic SCI with no differences between male and female. **G-H** Assessment of depression-like behavior with novelty suppressed feeding (NSF) showed significant increase of latency time reach food in a novel arena (G) for SCI/M and SCI/F mice compared to Sham group, while SCI/F mice were even slower to reach the food in their respective home cages (**H**). **p < 0.01, ***p < 0.001 vs. Sham group; ^###^*p* < 0.001 vs. SCI/M group. Two-way ANOVA following Tukey’s multiple comparisons test. *n* = 13 for Sham groups, 13 for SCI/M, and 14 for SCI/F

Finally, we compared the functional outcomes of male and female mice after chronic SCI through behavioral assessment. The mortality rate for both sexes was significantly higher in SCI groups compared to the Sham surgery groups beginning at 12 months as determined by the Mantel–Cox test (Fig. 10A). Young female mice showed comparably less death than males at early timepoints of the injury but exhibited a stark reversal around 12mon post-injury. Thus, when examining the mortality rates as a whole from day 0 to 19mon, there are no sex differences within either Sham or SCI groups. Locomotor function of the hindlimbs were assessed by BMS, which showed no statistical significance between male and female in chronic SCI up to 82 weeks post-injury (*n* = 13/group, Fig. 10B). Assessment of cognitive and depressive-like behavior at endpoint (19mon) also did not yield significant differences between the two sex groups. Although aged SCI mice did not exhibit hippocampus-dependent spatial memory impairment in the Y-maze test as seen in percentage of spontaneous alternations, SCI markedly affected the total number of arm entries (Fig. 10C, D), indicating that injury has an impact on the mobility of mice after months of aging. These results can be corroborated with data derived from open field, which showed significantly lower distance travelled and movement speed (Additional file 1: Fig. S7A, B). We also found impairment in the NOR test for injury groups of both sexes; however, no sex differences were observed in either the sham or injury groups (Fig. 10E, F). Assessment of depressive-like behavior by NSF showed significant increase of latency times in a novel arena in the SCI groups, but no sex differences (Fig. 10G). In their home cages, we observed increased latency times to the food in SCI/F mice compared to SCI/M group (Fig. 10H). SCI mice in open field test showed significantly reduced distance in inner zone and increased time of freezing and immobility (Additional file 1: Fig. S7C–E). Taken together, cognitive impairment and significant loss of mobility persisted chronically in both sexes after chronic SCI and appears to be increased with advancing age. However, no sex differences were observed at the late stage of injury in either cognitive or motor functions.

## Discussion

Among the many long-term consequences of SCI, neuropsychological changes and neuropathology in the brain are poorly understood in those aging with SCI. To address this issue, we examined neuropathological alteration of the somatosensory cortex and hippocampus at 19mon after SCI. Transcriptional data showed that different pathways in the brain were activated chronically after injury depending on biological sex. Our findings provided evidence that aging changes after SCI are somewhat sex-dependent. Although the latest survey on SCI prevalence rates indicate young males as the predominant group at approximately 80% [28], the number of women living with an SCI is rising, with demographics shifting in recent years [47]. To date, most studies examining long-term complications of SCI fail to address biological sex as a potential factor. The present study is the first to examine the impact of sex and aging on spinal cord and brain transcriptomic changes and EVs/EV cargo chronically after experimental SCI in mice.

We have previously reported on transcriptome data of the cerebral cortex from middle-aged mice (6mon of age) at 8mon post-injury and found far fewer sexually dimorphic genes in the neuroinflammation panel [16]. We speculated that the lack of sexual differences might reflect age at the onset of injury, which was close to perimenopause age, with age of euthanization occurring during a period of reproductive senescence [48]. Thus, the time of injury in the current study was changed to 10–12 weeks of age, which is considered young adult for C57BL/6 mice. Furthermore, due to the chronic attenuation of neuroinflammatory response that we had previously reported [16] and because several studies had reported on an endogenous shift towards a proinflammatory response in the brain with advanced age [49, 50], we used the Neuropathology panel in the current evaluation.

As noted in our results, there are far more robust changes to gene expression when comparing naturally aged mice to mice aging after SCI. Moreover, there are a larger number of DEGs in the somatosensory cortex than the hippocampus. These results are consistent with our previous findings, showing that neuroinflammation was chronically elevated by SCI in both the lesion site and the cerebral cortex. The high number of genes associated with activated microglia may support the concept that depression and cognitive deterioration chronically after SCI reflect, in part, microglial-mediated chronic inflammation [36]. Examination of primary sex effects in the neuropathology panel also showed many more DEGs in the cerebral cortex (147 genes for sham, 185 genes for SCI) than we had previously reported for middle-aged mice (24 for sham, 59 for SCI). For the genes of interest that we isolated from the top DEGs of SCI/F vs. SCI/M, specific functions and pathways are suggestive of regional dependency. In the somatosensory cortex, many gene changes are associated with regulation of axon and dendrite structure. Examples include the genes *Mtor*, which is a major regulator of axonal growth [51–53]; *Dlg4,* which encodes the protein PSD95 [54, 55]; *Grin1*, which encodes a critical subunit of NMDA receptors [56, 57]; the neurofilament light chain gene *Nefl*, which is being developed as a biomarker in traumatic SCI and multiple sclerosis [58, 59]. Interestingly, the only gene that showed lower expression levels in SCI/F mice compared to SCI/M was *Adcy8*, which encodes Adenylate Cyclase 8, an enzyme that catalyzes cyclic AMP formation. This gene has been found to regulate short-term plasticity, presynaptic/post-synaptic LTP and anxiety-like behaviors induced by stress, with Adcy8-null mice showing less susceptibility to repeated exposures [60, 61].

In the hippocampus, the functions of the top 10 DEGs between the two injury groups are more varied, with 5 involved in microglial activation. Most notable are the sex differences in the genes *C1qa* and *C1qb*, which are both encoders of the serum complement subcomponent Complement Component C1q, an important molecule for innate immunity and microglia function. Multiple studies have shown a potential role of C1q in neurodegenerative disorders, such as AD, stroke and amyotrophic lateral sclerosis (ALS) [62–64], along with age-related macular degeneration [65].

The age of endpoint for the current study was seven months longer than we reported in our previous study [16]. The sex differences that we had observed by NanoString were indicative of sexual dimorphism being regulated by factors beyond sex hormones. A clinical study from Davis et al. examined whether X chromosome gene expression was associated with tau pathology by RNA sequencing [66]; they identified genes in the X chromosome that were significantly associated with cognitive change and tau pathology both males and females, along with 19 genes that were related to slower cognitive decline in women. Taken together, these studies corroborate our transcriptomic findings in the aging SCI brain.

Different brain regions show varying degrees of vulnerability to aging [67, 68]. The DEG differences across brain regions after SCI in male and female mice appear consistent with observations from Mangold et al*.* who examined genome expression in the hippocampus during aging (3 mon, 12 mon, and 24 mon). They found marked sex differences in the nature and magnitude of the aging responses, along with sexually divergent neuroinflammation that could contribute to age-related neurological diseases such as stroke and AD [50]. These authors also reported a sexual divergence between microglial-specific genes, including complement pathway components.

An interesting result from our flow cytometry analysis was the high number of microglia and myeloid cells observed in the female spinal cord at 19mon post-injury. Furthermore, SCI/F mice showed elevated levels of microglial ROS production in both lesion area and remote brain region. One of the most interesting findings from our study was significant sex differences in mitochondrial activity of brain microglia after long-term SCI. Previous work in AD shows that accumulation of dysfunctional mitochondria may exacerbate synaptic dysfunction and lead to increased occurrences of cognitive disorders [69]. Moreover, mitochondrial function in microglia has been found to play roles in the development of tau pathology in AD. These results further suggest that biological sex alters responses to aging with SCI.

EVs are nanoscale membrane vesicles with diverse origins that can be released into the extracellular microenvironment, carrying biologically active cargo and participating in cell-to-cell communication. We have previously characterized plasma EVs in a mouse model of acute SCI, which can cause remote inflammation in the brain [26]. However, no studies have thus far examined the effects of chronic SCI on circulating EVs in either sex. In the current study, we examined EVs derived from total plasma and tissue at 19mon post-SCI. The high level of tetraspanin EV biomarkers CD63 and CD81 in SCI/M mice suggested that certain EV subtypes may be elevated in males. By exploring the EVs of aged C57BL/6 mice in different sexes, Kim et al*.* found that the secretion of certain types of brain exosomes (Alix + and TSF101 +) increased during aging only in female mice, a change that may reflect aging and sex-dependent alterations in EV dynamics [70]. These findings support our hypothesis on biological sex playing a role in EV dynamics and potentially in functional recovery after SCI.

Of the two miRs that were decreased in the hippocampus-derived EVs of female mice (compared to males), *miR-124-3p* from neurons in the spinal cord has been found to be positively associated with disease severity in ALS [71], while *miR-34a-5p* was found to be an early biomarker of AD [72]. Another study found that *miR-124-3p* can attenuate neuropathic pain by targeting early growth response 1 (*Egr1*) [73], which coincides with one of the many DEGs that we were able to derive from our NanoString results. The downregulated *miR-34a-5p* was also reported to be increased in acute stroke and cognitive impairment induced by irradiation [74, 75]. More recent publications showed *miR-34a-5p* playing a role in inflammation and neuronal cell death in a rat model of SCI [76]. More significant changes were observed in the EV miRs that exhibited an increase in female mice compared to males. The highest fold change was observed in *miR-21-5p*, which increased as many as 3.52 times in female mice compared to males. This miR has been known to promote microglia polarization into the proinflammatory M1 form [77]. Although there are also contradicting studies that show *miR-21-5p* in a neuroprotective light [78, 79], it is generally agreed that *miR-21-5p* is involved in stress signaling and pathologic settings [80]. Collectively, these results suggest that pathologic changes in the hippocampus could worsen with age in female mice. But there were no significant cognitive changes chronically across sexes.

In the spinal cord, we found that several of the miRs that displayed sex main effects were part of the *let-7* family (*let-7f-5p, miR-98-5p, let-7g-5p, let-7b-5p*), which are highly conserved between species and play a critical role in regulating cell cycle and cellular differentiation [81], along with control of innate immunity and release of major cytokines [82]. Furthermore, several publications showed that *let-7* contributes to neurotoxicity and neurodegeneration in the brain [83, 84], which is further supported by clinical data that found elevated levels of *let-7b* and *let-7e* in the cerebrospinal fluid of Alzheimer’s disease patients [85]. Closer examination of the miRs changed by chronic SCI in our study showed increased expression of *let-7b* in both the hippocampus and spinal cord, while *let-7d* was significantly elevated in the plasma and cortical tissue derived EVs. Consequently, these reports seem to coincide with our earlier findings on cell cycle activation and cognitive impairment in both mice and rats [14, 15]. One possible mechanism could be increased release of EVs containing *let-7* that cause cell cycle dysregulation in the brain which then promotes neuropathology. The elevated levels of *let-7* miRs in EVs at 19mon post-injury may also explain why SCI/F mice exhibited worse mortality rates at very late timepoints, even though they showed clear neuroprotection at 13 weeks post-injury in our previous article [16]. If altered tissue and circulating EV *let-7* miRs directly contribute to SCI-mediated mortality and neurological functional impairment require further examination.

## Conclusion

We found that biological sex plays affect in the regulation of neuropathological transcriptomes in distal brain regions after a single isolated traumatic contusion injury to the spinal cord. This study is the first to examine SCI transcriptomic changes in brain long after SCI in association with aging. Moreover, we demonstrated that plasma- and tissue-derived EV concentration and cargo differ as a function of sex after SCI, which may contribute to longer-term functional changes. Thus, this study sets the stage for future quantitative neuropathological analyses and more extensive late cognitive assessments. In addition, assessment of blood pressure, blood glucose, overall activity level, sleep conditions, circadian cycles are reflective of the post-traumatic recovery process, that remain an intriguing area for future investigation.

### Supplementary Information

**Additional file 1:** Supplemental Figures.

## Data Availability

All data needed to evaluate the conclusions in the paper are present in the paper and/or the Supplementary Materials.
