# Supplementary material for: Sexually dimorphic extracellular vesicle responses after chronic spinal cord injury are associated with neuroinflammation and neurodegeneration in the aged brain
Source: J Neuroinflammation. 2023 Aug 31;20:197. doi: 10.1186/s12974-023-02881-z (PMC10469550; doi:10.1186/s12974-023-02881-z)
Supplement: Supplementary file 1 — Additional file 1: Supplemental Figures. [file 12974_2023_2881_MOESM1_ESM.pdf]

Sexually dimorphic extracellular vesicle responses after chronic spinal cord injury are associated with neuroinflammation and neurodegeneration in the aged brain

Yun Li[1], Niaz Khan[1], Rodney M. Ritzel, Zhuofan Lei, Samantha Allen, Alan I. Faden, Junfang Wu*

Department of Anesthesiology and Center for Shock, Trauma and Anesthesiology Research (STAR), University of Maryland School of Medicine, Baltimore, MD, 21201 USA.

[1]Y.L. and N.K. contributed equally to this work.

*Correspondence to Dr. Junfang Wu, University of Maryland School of Medicine, 685 W. Baltimore Street, MSTF, Room 6-034D, Baltimore, MD 21201 USA; Tel: +1 410 706 5189. E-mail address: junfang.wu@som.umaryland.edu

Supplementary Information

Supplemental Information includes Supplemental seven figures and figure legends.

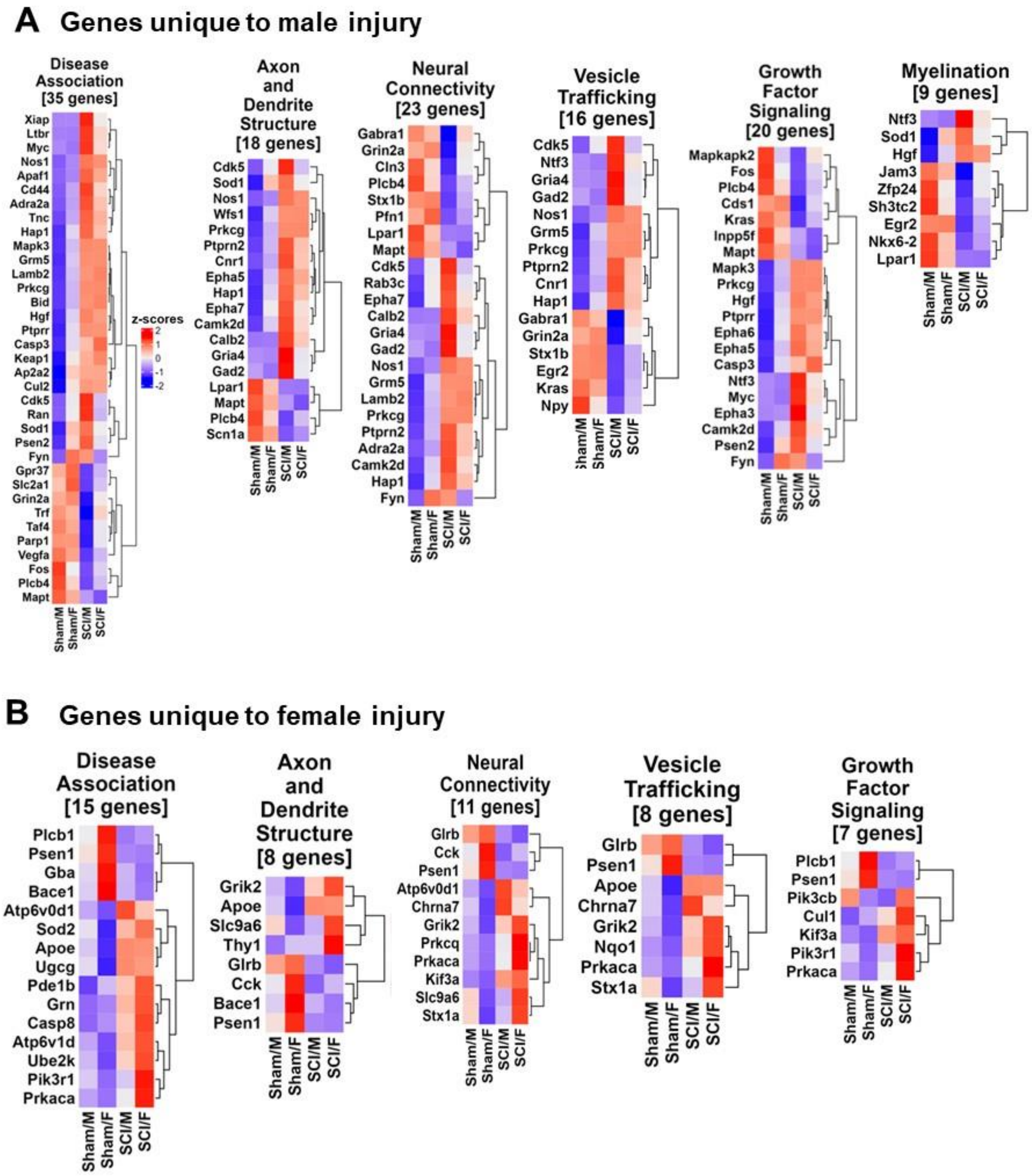

Supplementary Figure 1. Chronic SCI alters different genes associated with neuropathology in male and female. Expression profile of genes are related to disease association, axon and dendrite structure, neural connectivity, vesicle trafficking, growth factor signaling and myelination. **(A)** Heatmap for male specific DEGs induced by SCI. **(B)** Heatmap for female specific DEGs induced by injury. n=5-6 mice/group. Color coding is based on z-score scaling.

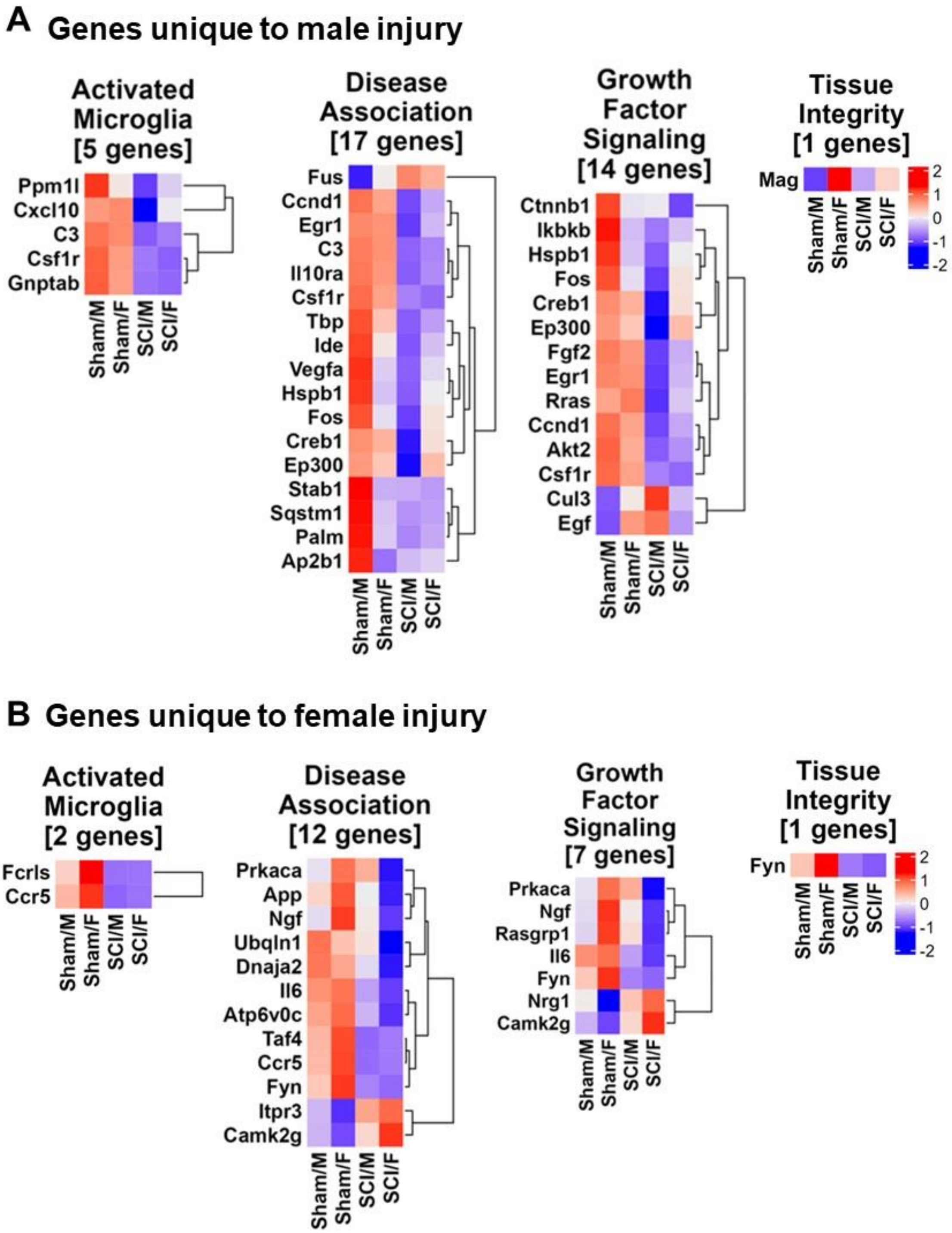

Supplementary Figure 2. Sex dimorphism induces unique injury genes in male and female mice at 19mon SCI. Expression profile of genes related to activated microglia, disease association, growth factor signaling and tissue integrity. **(A)** Heatmap of genes unique to male injury. **(B)** Heatmap of genes unique to female injury. n=5-6 mice/group. Color coding is based on z-score scaling.

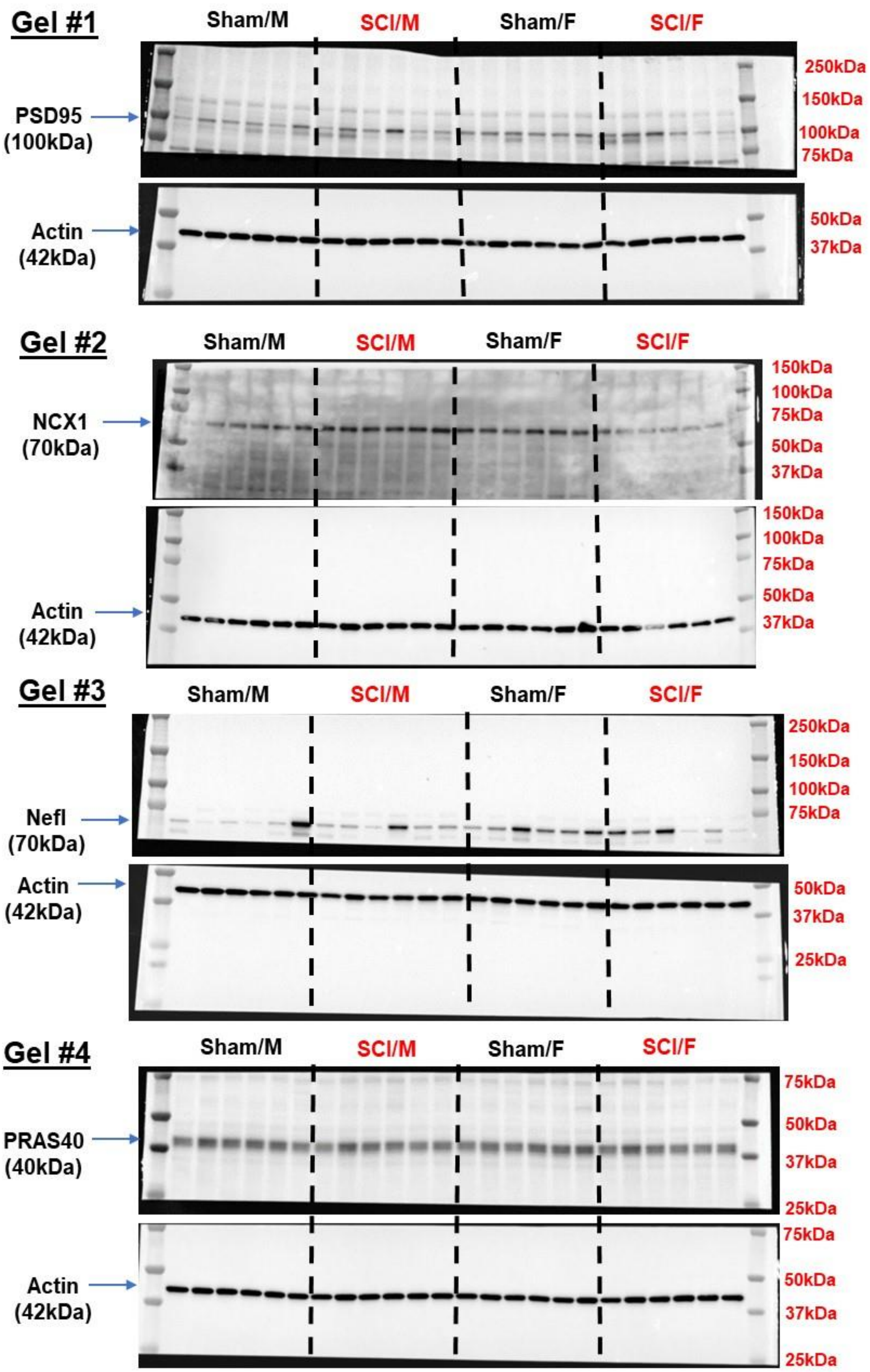

Supplementary Figure 3A. Unedited cortex western blots of Figure 5A.

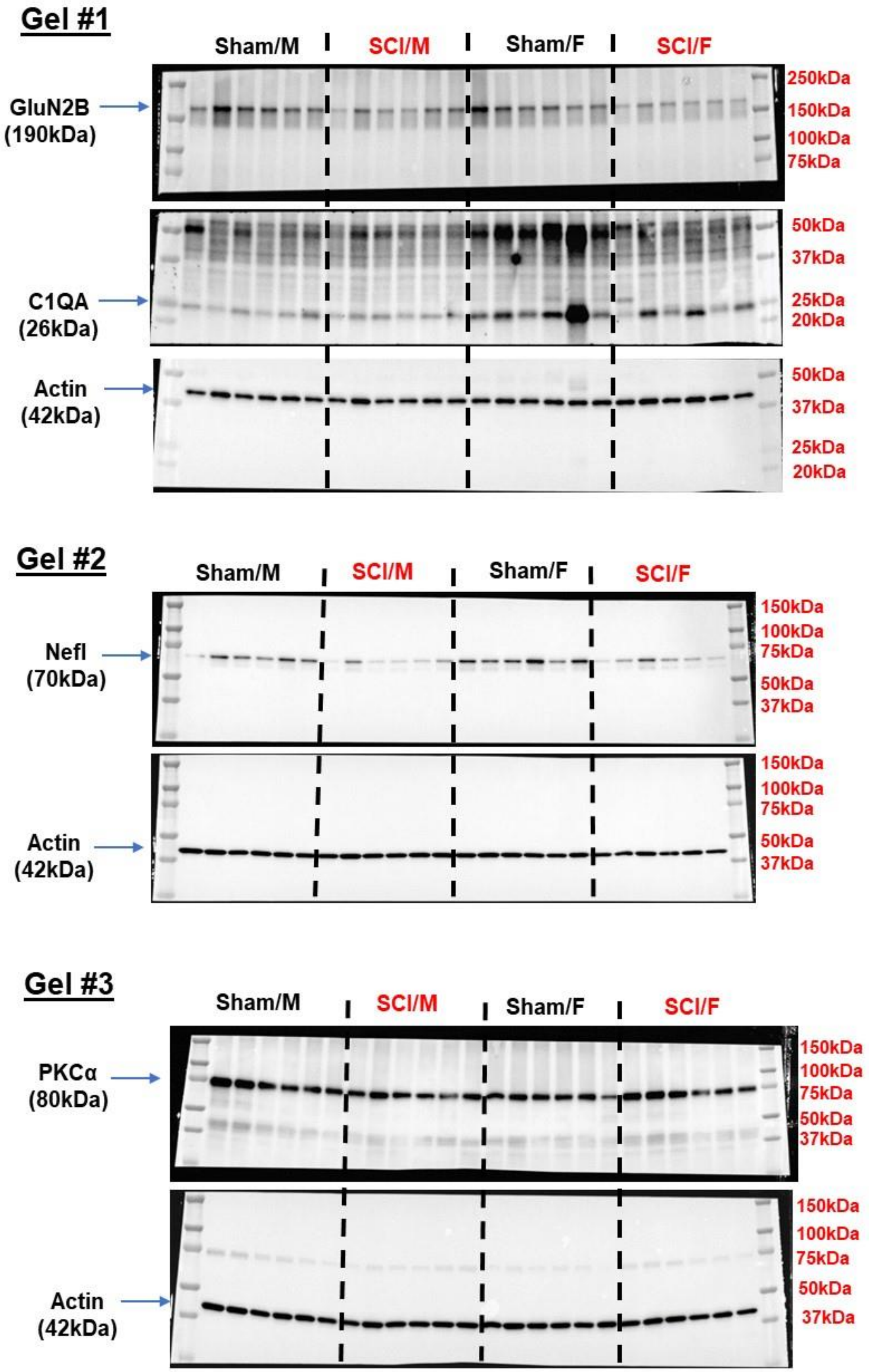

Supplementary Figure 3B. Unedited hippocampus western blots of Figure 5B.

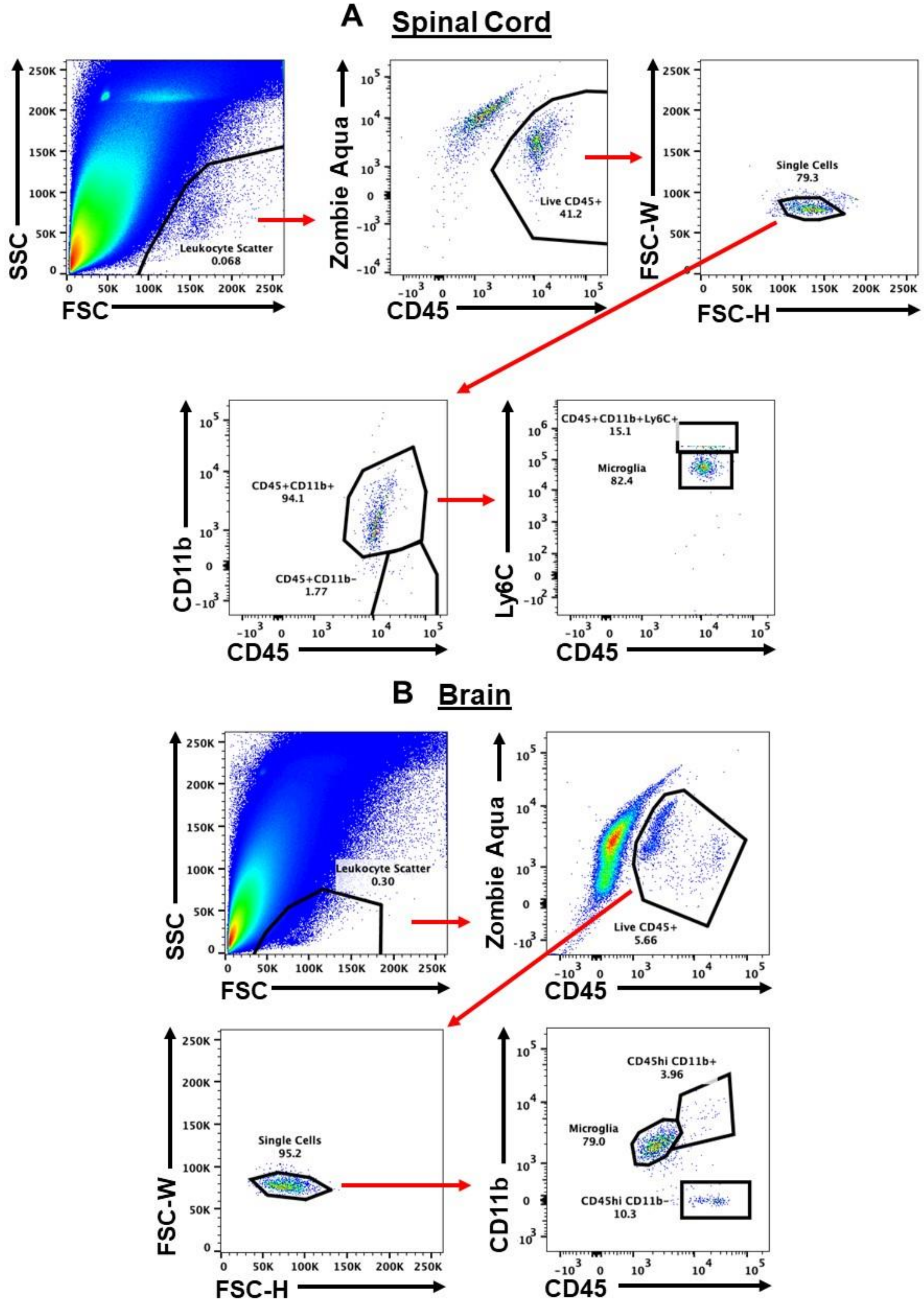

Supplementary Figure 4. Representative gating strategy in flow cytometry. For identification of immune cell composition in the spinal cord (**A**) and the brain (**B**).

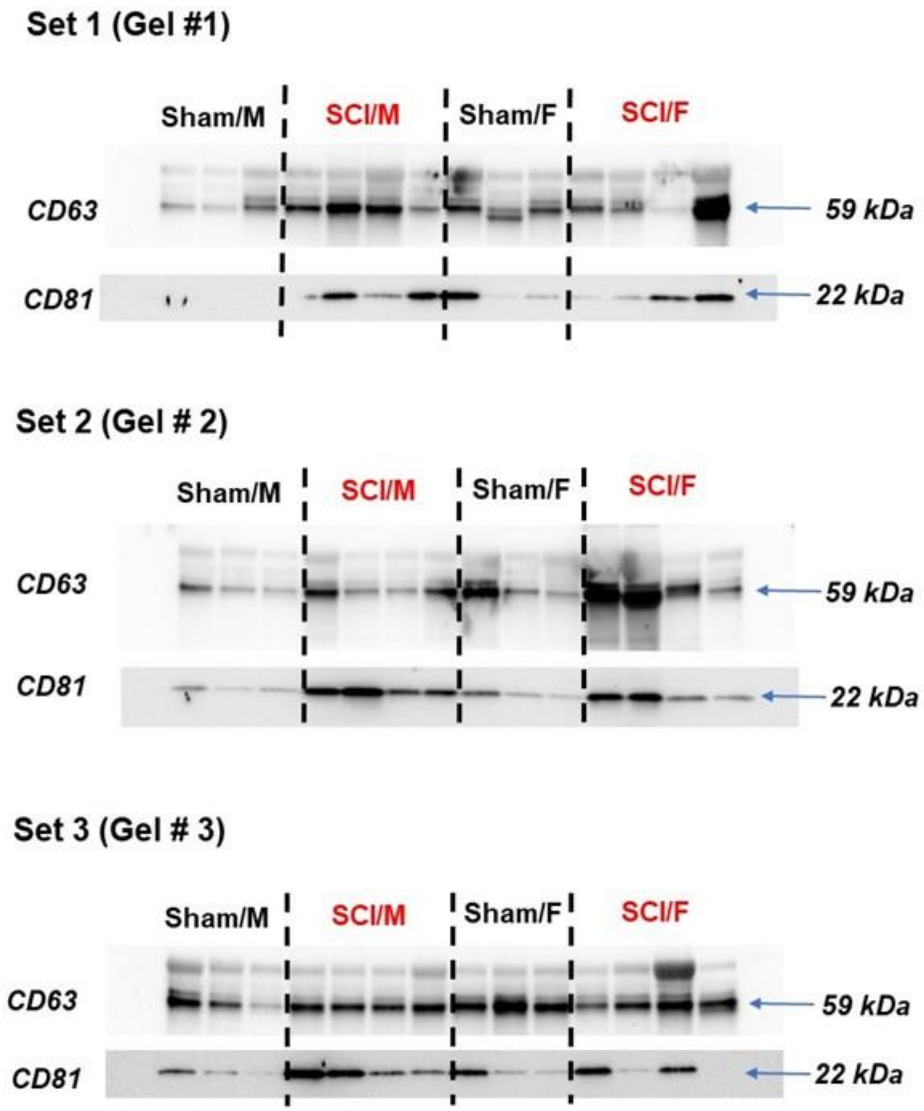

Supplementary Figure 5. Unedited EVs western blots of Figure 8A-B. Each lane in each set/gel represents an individual sample. n=9 (Sham/Male), 12 (SCI/Male), 9 (Sham/Female), and 12 (SCI/Female) mice. Western blot analysis was performed by loading an equal volume of isolated EVs. This is a common practice for characterization of EV markers with Western Blot, as the often-used housekeeping protein of GAPDH or ACTIN were practically undetectable in the EVs samples. In the guidelines [Thery, C., et al., Minimal information for studies of extracellular vesicles 2018 (MISEV2018): a position statement of the International Society for Extracellular Vesicles and update of the MISEV2014 guidelines. J Extracell Vesicles, 2018. 7(1): p. 1535750)], no recommendations were made for selection of a housekeeping protein that is stably expressed in all EVs and can be used for Western blotting.

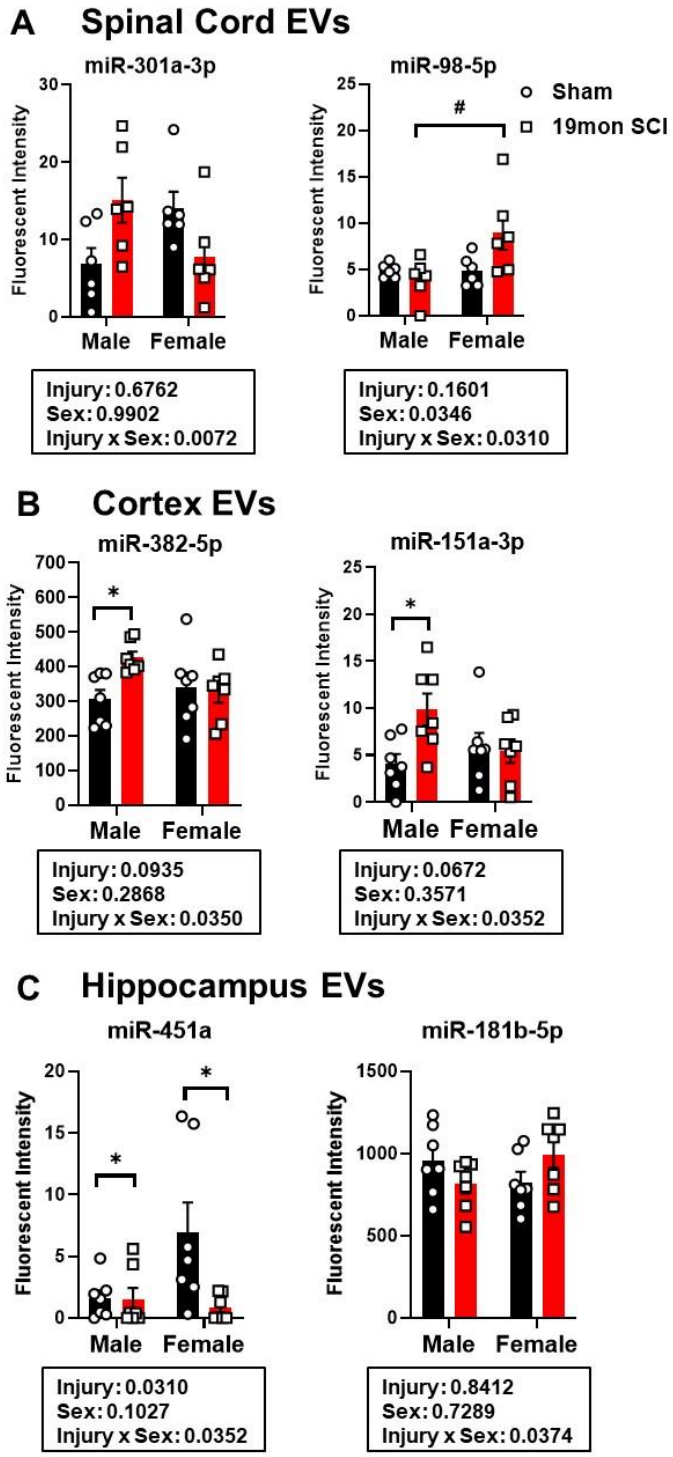

Supplementary Figure 6. Expression levels of miRs with an interaction effect between sex and injury. Normalized fluorescent intensity of the miRs detected in EVs derived from the spinal cord (**A**), the somatosensory cortex (**B**) and the hippocampus (**C**) are shown as mean and SEM with individual data points. n=6/group for spinal cord EVs, n=7/group for cortex and hippocampus EVs. *p<0.05 vs. Sham group, #p<0.05 vs. SCI group. Two-way ANOVA followed by Tukey's multiple comparisons test.

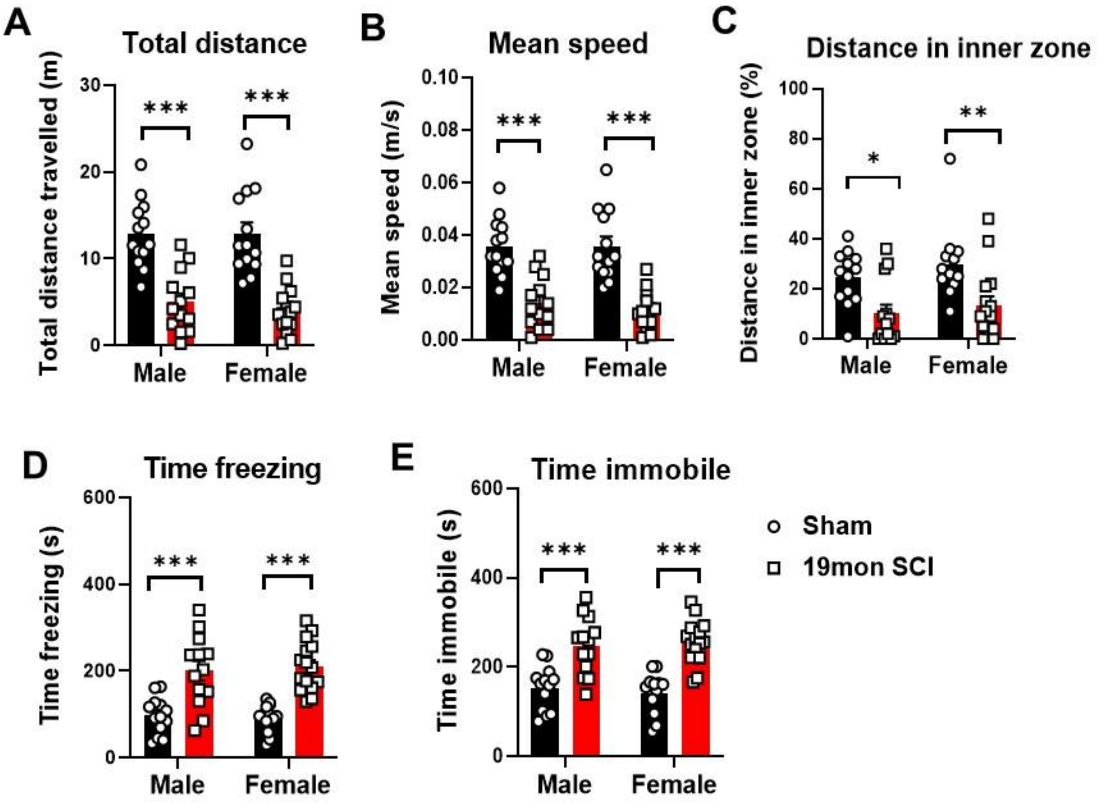

Supplementary Figure 7. Age mice show decreased spontaneous activity and anxiety-like behavior in open field test at 19 months post-injury. Injury effects were observed in five parameters: total distance (**A**), mean speed (**B**), distance travelled in the inner zone (**C**), time freezing (**D**), and time immobile (**E**). *p<0.05, **p<0.01, ***p<0.001 vs. Sham group. n=13 for Sham groups, 13 for SCI/Male, and 14 for SCI/Female. Two-way ANOVA following Tukey's multiple comparisons test.
